# Promelaxin Microenemas Are Non-inferior to Oral Polyethylene Glycol for the Treatment of Functional Constipation in Young Children: A Randomized Clinical Trial

**DOI:** 10.3389/fped.2021.753938

**Published:** 2021-10-29

**Authors:** Caterina Strisciuglio, Vincenzo Coppola, Marina Russo, Carlo Tolone, Gian Luigi Marseglia, Alberto Verrotti, Silvia Caimmi, Claudia Caloisi, Valeria D'Argenio, Lucia Sacchetti, Annamaria Staiano

**Affiliations:** ^1^Department of Woman, Child and General and Specialized Surgery, University of Campania “Luigi Vanvitelli”, Naples, Italy; ^2^Department of Translational Medical Sciences, Section of Pediatric, University of Naples Federico II, Naples, Italy; ^3^Maternal and Child Department, IRCCS Foundation Policlinico “S. Matteo” di Pavia, Pavia, Italy; ^4^Department of Pediatrics, University of Perugia, Perugia, Italy; ^5^Department of Pediatrics, University of L'Aquila, L'Aquila, Italy; ^6^CEINGE Biotecnologie Avanzate S. C. A R. L., Naples, Italy; ^7^Department of Human Sciences and Promotion of the Quality of Life, San Raffaele Open University, Rome, Italy

**Keywords:** polyethylene glycol, Promelaxin microenemas, medical devices based on substances, functional constipation, young children

## Abstract

**Background:** Polyethylene glycol (PEG) is recommended as first-line treatment of pediatric functional constipation. However, the oral route of administration is often poorly feasible in children mostly due to poor palatability. Promelaxin microenemas exert a topical evacuative action and may offer a valuable option in pediatric FC.

**Aim:** To assess whether Promelaxin microenemas would be non-inferior to PEG 4000 in young children with FC.

**Methods:** This is a randomized, open-label, multi-centric, non-inferiority trial enrolling infants and young children aged 6–48 months, with FC according to Rome III criteria. After 1 week of run in, children were randomized to 2 weeks of Promelaxin or PEG, followed by a 6-week on-demand treatment period. Primary endpoint was response rate to randomized treatment, with “response” defined as at least 3 evacuations per week and an average increase of at least one evacuation per week as compared to baseline. Safety, stool consistency and the analysis of fecal microbiota were secondary endpoints.

**Results:** Out of the 158 patients who entered the trial, 153 patients were treated (77 and 76, PEG and Promelaxin arm, respectively). In the primary analysis, the 95% confidence interval (CI) for the treatment's effect lay entirely above the non-inferiority margin in both Full Set (FAS) and Per Protocol (PP) analyses, providing evidence of the non-inferiority of Promelaxin vs. PEG 4000 [response rate difference: 16.5% (CI 1.55–31.49%) and 11.03% (CI −5.58 to 27.64%), FAS and PP analyses, respectively]. Mean compliance to the randomized treatment was >80% in both arms. Secondary endpoints did not significantly differ between the two arms, except for the average number of total days of on-demand treatment that was significantly lower in the Promelaxin arm [14.6 (12.7) vs. 9.8 (9.1), mean (SD); primary endpoint responders in PEG and Promelaxin arm, respectively; *p* = 0.027]. Microbiota evenness significantly increased in the PEG 4000 arm at V4 as compared to the Promelaxin arm (*p* < 0.05). In addition, at V5, patients treated with PEG showed a significantly decreased microbiota density as compared to patients treated with Promelaxin (*p* = 0.036).

**Conclusions:** Promelaxin microenemas are non-inferior to oral PEG in children with FC.

**Clinical Trial Registration:** www.ClinicalTrials.gov, identifier: NCT02751411.

## Introduction

Functional constipation (FC), defined as the infrequent (<2/week) and painful passage of stools associated with stool retention, is a common problem in childhood (1, 2). A 2018 systematic review and meta-analysis reported the worldwide pooled prevalence of FC in children to be 9.5% (95% CI 7.5–12.1%) (2, 3). Overall, 1 in 10 children may suffer from FC. The transition to solid food, toilet training and school entry are usually precipitating events associated with the onset of FC (3, 4).

In infants and toddlers, FC usually appears to originate from an acquired behavior of stool withholding after experiencing painful defecation (4). This makes the rectal fecal mass difficult to eliminate, thus amplifying the persistence of constipation. In order to avoid long-term FC, a successful integrated treatment strategy should be implemented at an early stage, combining pharmacological and non-pharmacological approaches (4). In fact, it is recommended to integrate early pharmacological treatment with non-pharmacological treatment, i.e., behavioral, psychological, dietary interventions (2, 5, 6), to interrupt the loop leading to the persistence of FC, fecal impaction, psychological problems, and a significant burden on children and parents (7). Considering that the median age for the onset of this condition is around 2 years (8), acting before or around that age becomes crucial, in order to interrupt the vicious loop leading to persistent FC and to avoid its complications, such as fecal impaction.

Oral polyethylene glycol (PEG) is currently recommended as the first-line treatment of pediatric FC by the major international Societies (9). However, adherence to PEG can be sub-optimal especially in infants, often due to poor palatability (10) as reported by parents. Consistently, adherence as low as 37% has been reported in children on long-term treatment with PEG for persistent FC (11). Therefore, other treatment options with an efficacy comparable to PEG could be relevant in optimizing the treatment of FC, especially in young children.

Enemas are used in pediatric patients, with volumes adapted to the function (local or systemic effect) and to the age of the child (7, 12). A randomized trial in children suffering from fecal impaction, a condition that, triggers or complicates FC, compared oral PEG vs. enemas (60 ml of dioctyl sulfo-succinate-sodium, once-daily for 6 days) in children aged 4–6 years. The trial showed that enemas were as effective as high-dose oral PEG (13).

Therefore, we tested whether also in the case of FC, an early and short-term treatment with microenemas (4 ml volume) might be as effective as oral PEG in infants and toddlers, and thereby possibly become a treatment option. In addition, since osmotic laxatives have been shown to be associated with changes in the gut microbiota in studies in humans as well as in animal models (14–18), we also tested whether microenemas, through their topical effect, may have a lesser effect on the gastrointestinal microbiota. Thus, we performed a randomized comparison between oral PEG and microenemas containing honeys and polysaccharides.

## Materials and Methods

### Study Design and Patients

This is a randomized, controlled, open label, multicenter, non-inferiority trial aimed at evaluating the efficacy and safety of Promelaxin microenemas (Melilax Pediatric, 4 ml/5 g, volume/weight, Aboca, Sansepolcro, Italy), vs. PEG 4000 (Paxabel 4 g, Ipsen Consumer Healthcare S.r.l., Milano, Italy) in the short-term treatment of FC. Promelaxin is a CE marked class IIb medical device made of 100% natural substances, which exerts a local evacuative action. Promelaxin microenemas are marketed in Europe. The study was carried out in four primary care hospitals in Italy between April 2016 and March 2020. The study protocol was approved by the local Ethical Committees of each participating center (coordinating center approval date: 09/09/2015, approval number 190/15) and was conducted in accordance with the Declaration of Helsinki. The written Informed Consent to participation in the study was obtained from the parents of all patients prior to their enrollment. The study was registered in Clinicaltrial.gov (NCT02751411) and EudraCT Database (2015-005111-32).

Inclusion criteria were male and female children aged from 6 to 48 months with a diagnosis of FC according to the Rome III criteria (19). Exclusion criteria were: suspicion or diagnosis of organic diseases causing constipation such as inflammatory bowel disease, motility disorders, neurological disorders, inherited and metabolic disorders, surgical disorders, anal fissures and Hirschsprung's disease. Treatments with fecal softeners or prokinetics, probiotics, prebiotics, herbal dietary supplements or other herbal products and psychiatric drugs were all forbidden during the trial, starting from the run-in. The 7-day run-in phase was instrumental in ruling out the presence or development of fecal impaction prior to randomization. Parents were provided with dietary recommendations regarding fiber intake at enrollment, as well as recommendations on appropriate toilet training (9).

**Figure 1:**
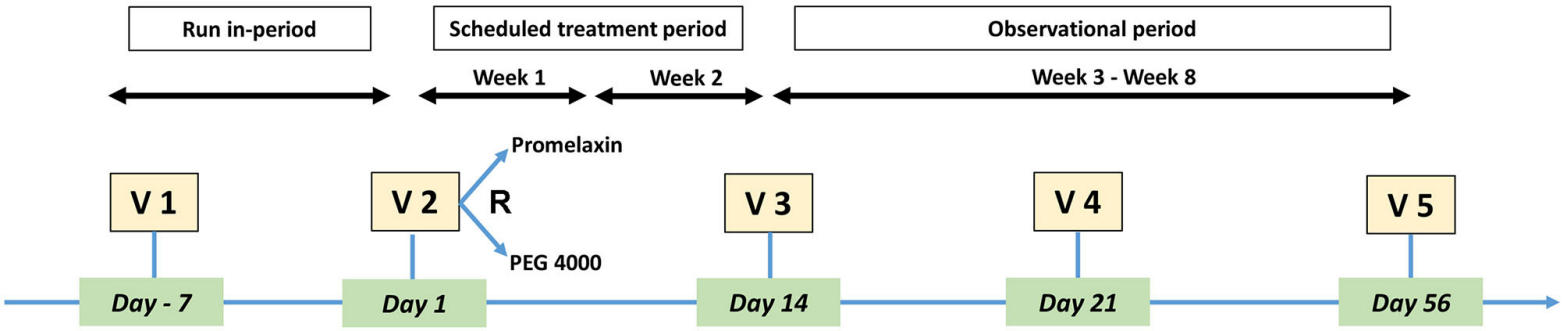
Study Design. The figure depicts the design of the study, including study Visits.

The study design is shown in Figure 1 and included: a 1-week run-in, a 2-week randomized treatment, followed by a 6-week observational period during which randomized treatment could be repeated on-demand, with 5 study visits in total. At visit 2 (V2), eligible patients were randomly assigned (1:1) to PEG or Promelaxin, according to a predefined block randomization list. Each center opened the randomization letters in sequential order to randomize patients. On demand treatment was defined as the need for PEG or Promelaxin (according to the randomized arm) after 48 h without a fecal evacuation. After V3, when primary endpoints were evaluated, two more visits were scheduled at day 21 (V4) and day 56 (V5 and end of study visit).

For each study period (run-in, treatment, on-demand period) a daily diary was supplied to parents to record the number of evacuations, the consistency score of stools, the administration frequency and dosage of the Investigational Product (IP), any associated gastrointestinal symptom (regurgitation, vomiting, flatulence, lack of appetite, diarrhea), any other symptom they deemed relevant, and any concomitant medication, meaning any medication other than the treatment in the study. Stool consistency was scored according to the Amsterdam Stool Form Scale (ASFS) (20) for children under 1 year of age and according to the Bristol Stool Form Scale (BSFS) (21) for children over 1 year of age.

A quality of life (QoL) questionnaire (extrapolated from PedsQL) (22) and parent QoL (Visual Analog Scale, VAS) were also filled in at study visits, to assess safety and treatment compliance and the most recent stool consistency score was registered by the Investigators.

The daily dosage of Promelaxin was 2.5 g (2 ml, half a microenema) for children 6–12 months old, 5 g (4 ml) for children aged 12–48 months; children 36–48 months were allowed a maximum of 10 g (8 ml). The daily dosage of PEG 4000 was decided by the Investigator based on the child's body weight and approved posology (i.e., 4 g/day for children 6–12 months old and 4–8 g/day for children 12–48 months old). During the 2-week randomized period (between V2 and V3), IPs were administered every day for the first week and every second day in week 2. During the on demand period (weeks 3–8), the same daily dosage was administered but on-demand. No other laxative/microenemas other than the IPs were allowed during the entire study.

### Primary and Secondary Endpoints

The primary endpoint was stool frequency expressed as response rate assessed at V3, with “response” defined as 3 or more evacuations per week and an average increase of at least one evacuation per week as compared to the run in (baseline) in case of ≥3 evacuations at baseline. as reported on the patients' daily diary.

Secondary endpoints included: response assessed as stool consistency, with responders defined as patients who experienced an increase, as compared to baseline, of one or more points on the ASFS or BSFS; “normalization” of bowel habits as defined by a composite response of stool frequency and consistency; days with gastrointestinal symptoms; intestinal microbiota profile; QoL scores, measured through a VAS scale ranging between 0 mm (Very good) and 100 mm (Very bad). A reduction from baseline of at least 1 point was considered as a QoL improvement.

### Microbiota Sub-study

Instructions for fecal sample collection were given to the parents at V1. Fecal samples were collected at V2, V4, and V5 and stored at −80°C until analyzed.

Microbiota analysis was performed targeting the 16S rRNA gene variable region V4-V6 (23). DNA extraction, library preparation and sequencing were performed according to the protocol described by Nardelli et al. (24) 16S rRNA sequencing was performed using MiSeq Illumina sequencing (PE 2 × 300 cycles). The resulting 16S rRNA fastq files were analyzed using a standard procedure with Qiime 2.0 platform (25). After the overlapping and quality control of forward and reverse sequencing reads, the forward fastq files were used for further analysis (24). The feature table was constructed using the DADA2 algorithm integrated in Qiime 2.0 pipeline, keeping an average of ~59,000 reads per sample. The Pielou index was used to measure bacterial evenness, rarefacting the feature table at 24,000 reads per sample. Pielou's index measures diversity along with species richness. While species richness is the number of different species in a given area, evenness is the count of individuals of each species in an area. A calculated value of Pielou's index ranges from 0 (no evenness) to 1 (complete evenness). DNA concentration (ngDNA/100 mg fecal sample) was used to identify microbiota density (14).

### Sample Size Calculation

The sample size was calculated assuming a non-inferiority margin of 15% for the difference of the response rate for stool frequency between the two treatments assessed at V3. A response rate to PEG 4000 up to 93% was anticipated based on the available literature. Considering a drop-out rate of 25%, 80 patients per arm would achieve 80% power to detect non-inferiority with a one side significance level (alpha) of 0.025.

### Statistical Analysis

The following analyses were considered: Full Analysis Set (FAS) which considered all randomized subjects who received at least one dose of the IP; per protocol (PP) analysis that considered all subjects in the FAS population with no major protocol deviation until V3 with an evaluable primary endpoint. Microbiota analysis included patients in the FAS population with available fecal samples. The safety analysis included all randomized subjects who received at least one dose of the IP.

In the FAS analysis, patients with no data for primary endpoint assessment (i.e., baseline data missing, patient diary not returned) were considered non-responders. For the primary endpoint assessment, missing patient diary data were imputed using the worst-case approach. Comparison between arms was performed using *T*-test for continuous variables. Frequencies of Responders/Improved patients were compared using the Chi-square test. The difference was considered significant when *p* < 0.05, unless otherwise indicated. Differences in “counts” (e.g., no. of days of treatment use) were compared by using Poisson regression, adjusting for clinical site as a possible confounding factor. Kruskal-Wallis and *T*-Test were used to identify differences between arms of microbiota evenness and microbiota density, respectively.

The compliance to the IP was assessed using the information recorded in the patient's diary and was calculated as the ratio between treatment administered vs. planned. Compliance <70% was considered as a major deviation.

Descriptive statistics were used for demographics and baseline measurements.

## Results

**Figure 2:**
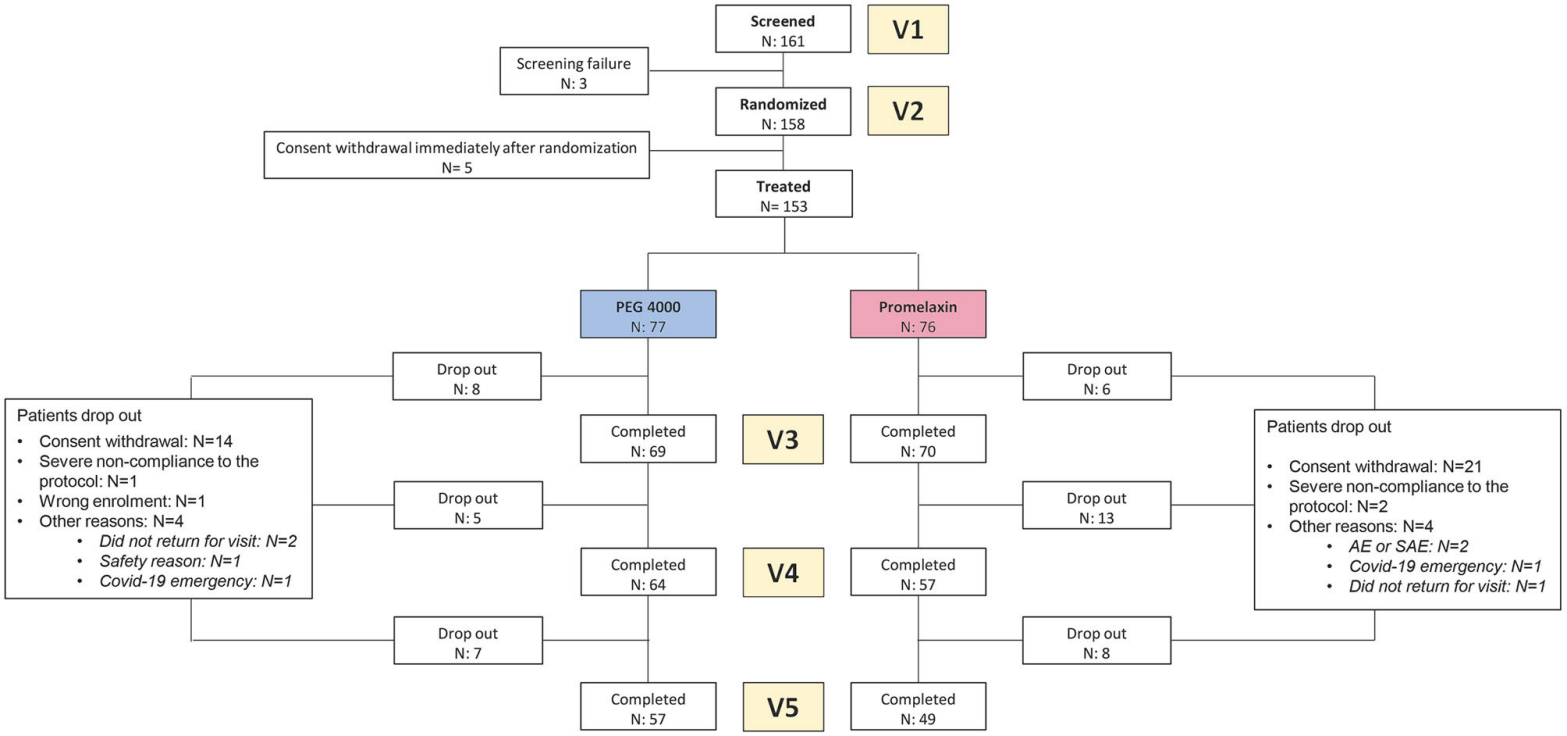
CONSORT diagram. The figure shows the Consort Diagram of the randomized trial.

One hundred and sixty-one patients were screened, and 158 patients were randomized. The consort diagram is shown in Figure 2. Patient's baseline demographics and clinical characteristics are summarized in Table 1, and no statistically significant differences were observed between the two treatment arms. Nearly 90% of the patients were studied at V3.

**Table 1:** Baseline and demographics (FAS population).

**Baseline parameters**	**PEG** **(*n* = 77)**	**Promelaxin** **(*n* = 76)**	***p*-value**
^*^General characteristics			
Age (days)	795.49 (400.17)	729.18 (342.87)	0.27
Height (cm)	88.22 (11.89)	86.37 (10.07)	0.32
Weight (Kg)	12.80 (3.00)	12.03 (2.67)	0.10
Gender (M)	32 (41.6%)	39 (51.3%)	0.2
Mother VAS (mm)	61.05 (26.69)	52.45 (33.42)	0.08
Father VAS (mm)	53.64 (28.03)	46.58 (33.20)	0.17
PedsQL total score	21.38 (3.71)	21.84 (3.09)	0.40
Stool frequency (week)	3.54 (3.11)	3.74 (3.34)	0.72
**ASFS – consistency (*****n*** **=** **25)**			
	*n* = 11	*n* = 14	
Type A, *n* (%)	1 (9.1%)	0 (0.0%)	0.58^**^
Type B, *n* (%)	1 (9.1%)	3 (21.4%)	
Type C, *n* (%)	3 (27.3%)	3 (21.4%)	
Type D, *n* (%)	6 (54.5%)	8 (57.1%)	
^*^BSFS – consistency (***n*** **=** **125)**			
	*n* = 64	*n* = 61	
Type 1, *n* (%)	19 (29.7%)	24 (39.3%)	0.70^**^
Type 2, *n* (%)	35 (54.7%)	32 (52.5%)	
Type 3, *n* (%)	6 (9.4%)	3 (4.9%)	
Type 4, *n* (%)	1 (1.6%)	0 (0.0%)	
Type 5, *n* (%)	0 (0%)	0 (0%)	
Type 6, *n* (%)	2 (3.1%)	1 (1.6%)	
Type 7, *n* (%)	1 (1.6%)	1 (1.6%)	

*Values are either Mean (SD) or n (%)*.

* *Some missing values. VAS, Visual Analog Scale; ASFS, Amsterdam Stool Form Scale for children aged <1 year; BSFS, Bristol Stool Form Scale for children aged ≥ 1 year*.

** *p-value is referred to the different distribution between treatment groups*.

**Figure 3:**
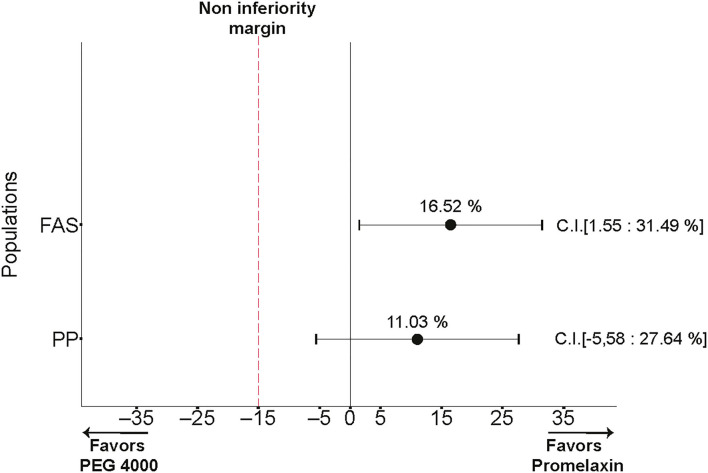
Primary endpoint. The differences in response rates between Promelaxin and PEG and relative 95% confidence interval (CI) are shown according to the full analysis set (FAS) and the PP analyses.

During the 2-week randomized phase, compliance was 84.32 ± 29.10% for PEG and 85.07 ± 25.23% for Promelaxin (mean ± SD, *p* = 0.87). As shown in Figure 3, the 95% CIs for the treatment effect lay entirely above the non-inferiority margin in both FAS and PP analyses, providing evidence of the significant non-inferiority of Promelaxin vs. PEG 4000 [response rate difference: 16.5% (CI 1.55–31.49%) and 11.03% (CI −5.58 to 27.64%), FAS and PP populations, respectively]. In addition, in the FAS, the proportion of responders was significantly higher in the Promelaxin arm as compared to PEG (72.4 vs. 55.8%, respectively; *p* = 0.033).

**Figure 4:**
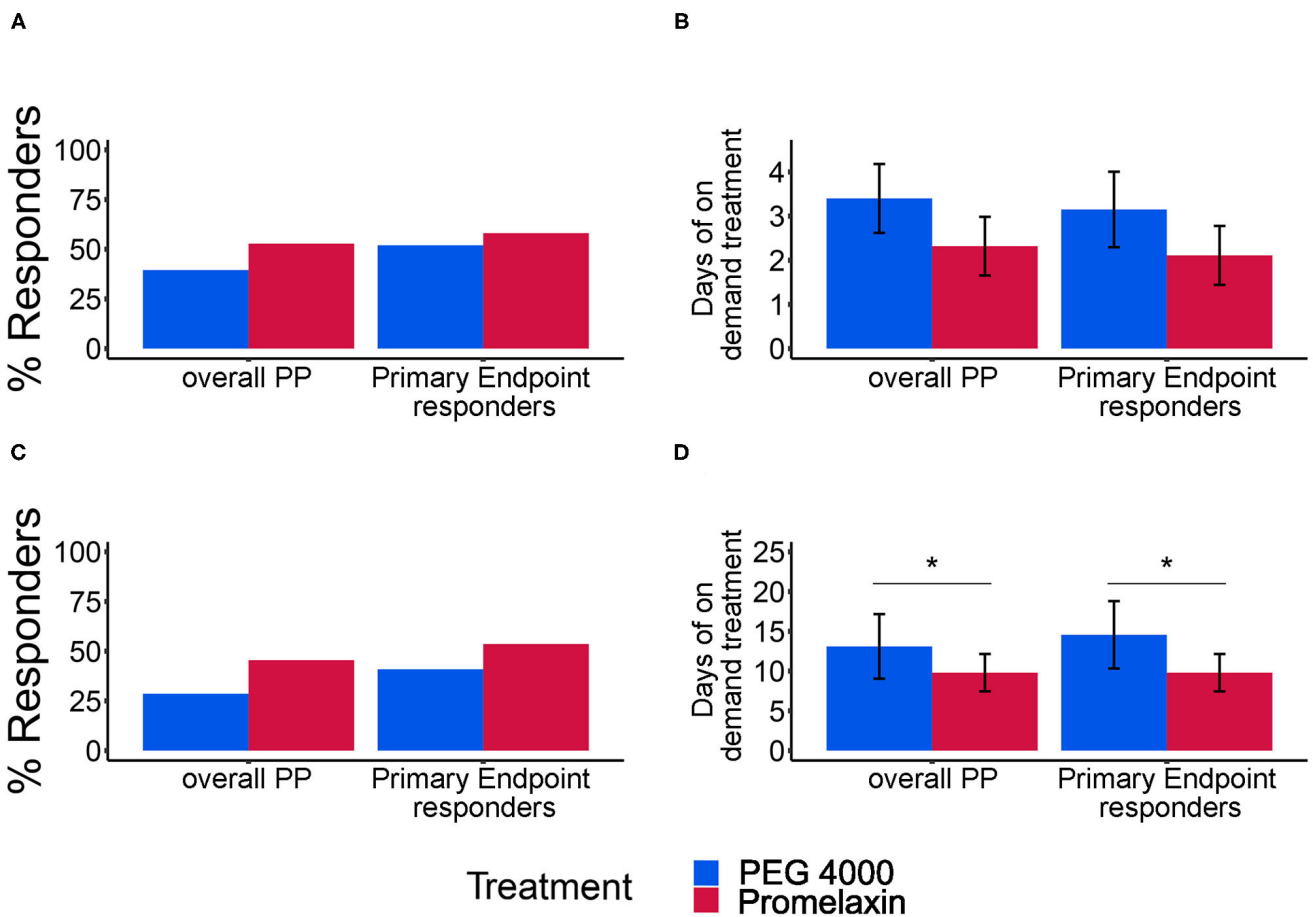
Response rate for combined stool frequency and consistency and average days of treatment. **(A,C)** show the response rate (%) for the combined stool frequency and consistency secondary endpoints at V4 and V5, respectively. **(B,D)** show the average days of treatment (mean ± SE) at V4 and V5, respectively. In each panel, results of separate analyses either according to the PP population or to the subgroup of PP patients evaluated as responders to the primary endpoint (Primary Endpoint Responders) are depicted. Error bars represent Standard Error. **(A)** Overall PP: PEG, *n* = 38; Promelaxin, *n* = 36; Primary Endpoint responders: PEG, *n* = 25; Promelaxin, *n* = 31. **(B)** Overall PP: PEG, *n* = 15; Promelaxin, *n* = 19; Primary Endpoint responders: PEG, *n* = 13; Promelaxin, *n* = 18. **(C)** Overall PP: PEG, *n* = 35; Promelaxin, *n* = 33; Primary Endpoint responders: PEG, *n* = 22; Promelaxin, *n* = 28. **(D)** Overall PP: PEG, *n* = 10; Promelaxin, *n* = 15; Primary Endpoint responders: PEG, *n* = 9; Promelaxin, *n* = 15. *p < 0.05.

Figure 4 displays composite response for stool frequency and consistency and the average days of treatment use, during and at the end of the observational period. At V4, 52.8% patients responded to Promelaxin compared to 39.5% who responded to PEG (Figure 4A, *p* = 0.25). This difference increased at V5, in favor of Promelaxin, although with a non-significant trend (Figure 4C, *p* = 0.14). The composite response rate showed the same trend when restricting the analysis to the responders to the primary endpoint (Figure 4, right columns).

At V4, the mean number of on-demand treatment days was lower in PP analysis of patients treated with Promelaxin (Figure 4B, *p* = 0.17). This difference became significant at V5 (Figure 4D; *p* = 0.02). Again, similar differences were observed when restricting the analysis to responders to the primary endpoint (right columns). In this latter subset, Promelaxin was used in 33% fewer days of the on-demand treatment period (*p* = 0.02, Figure 4). Stool frequency was also similar in the observation period, at V4 and V5 (*p* = 0.8 and *p* = 0.5).

**Figure 5:**
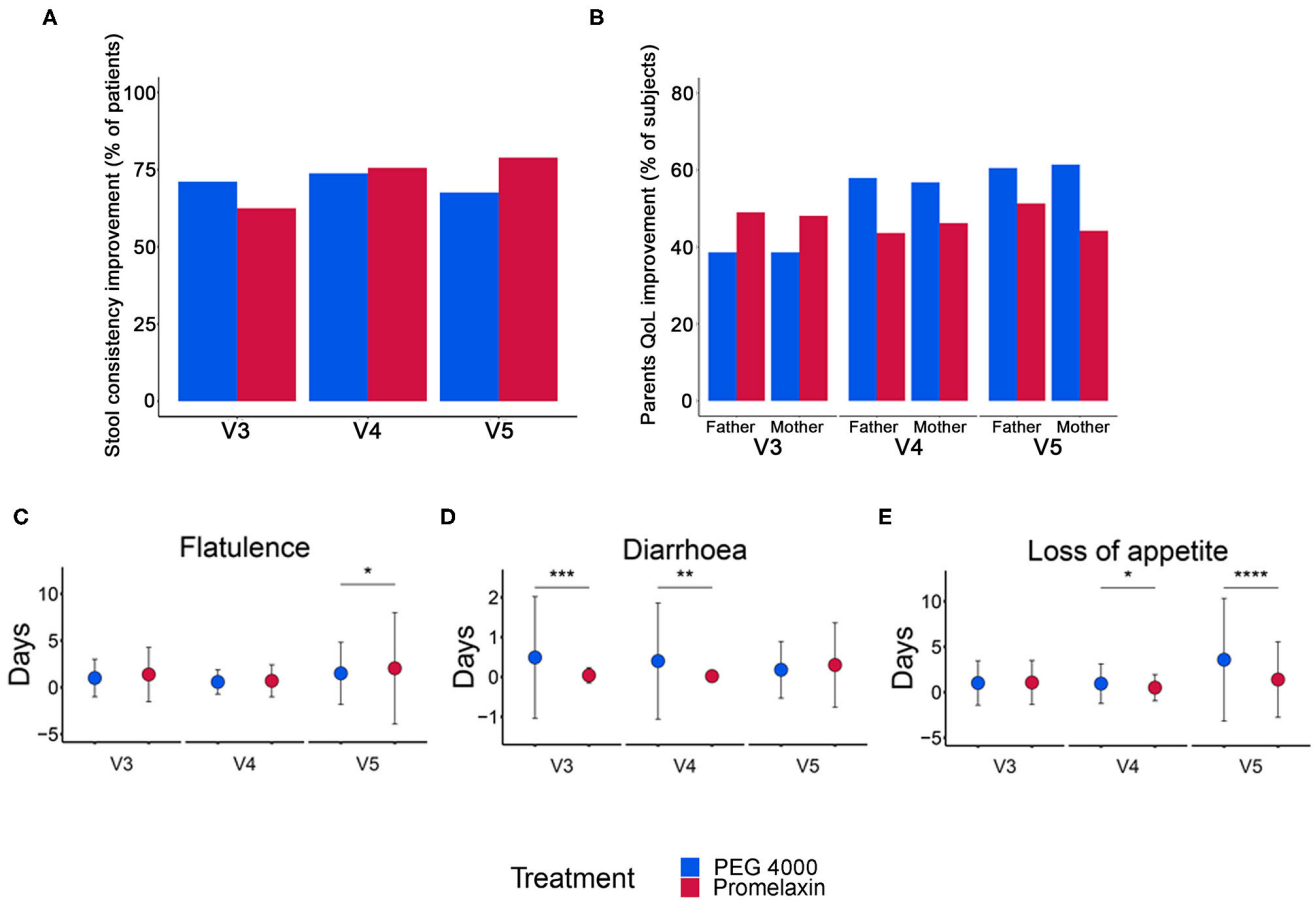
Stool consistency, QoL improvement and gastrointestinal symptoms in the randomized arms. **(A)** Percentage of patients with improved stool consistency at V3, V4, and V5. **(B)** Percentage of parents with improved QoL at V3, V4, and V5. **(C–E)** Number of days (mean ± SD) with gastrointestinal symptoms at V3, V4, and V5. Data are reported as mean ± SD. *p < 0.05, **p < 0.01, ***p < 0.001, and ****p < 0.0001. **(A)** V3: PEG, *n* = 45; Promelaxin, *n* = 56; V4: PEG, *n* = 42; Promelaxin, *n* = 45; V5: PEG, *n* = 37; Promelaxin, *n* = 38; **(B)** V3 (Father): PEG, *n* = 44; Promelaxin, *n* = 49. V4 (Father): PEG, *n* = 38; Promelaxin, *n* = 39. V5 (Father). PEG, *n* = 38; Promelaxin, *n* = 39; **(B)** V3 (Mother): PEG, *n* = 44; Promelaxin, *n* = 52. V4 (Mother): PEG, *n* = 44; Promelaxin, *n* = 52. V5 (Mother): PEG, *n* = 44; Promelaxin, *n* = 52. **(C–E)** V3: PEG, *n* = 45; Promelaxin, *n* = 56. V4: PEG, *n* = 40; Promelaxin, *n* = 47. V5: PEG, *n* = 40; Promelaxin, *n* = 47.

There was no significant percentage difference in improved stool consistency (PEG 4000 vs. the Promelaxin group 71.1 vs. 62.5%, *p* = 0.36; 73.8 vs. 75.6%, *p* = 0.85; 67.6 vs. 78.9%, *p* = 0.26; V3, V4, and V5, respectively; Figure 5A).

QoL was similar between the treatment arms at all time points (Figure 5B). The total number of days with gastrointestinal symptoms was low in both treatment arms (Figures 5C–E). Patients receiving PEG 4000 experienced significantly more days with diarrhea at both V3 (*p* < 0.001) and V4 (*p* < 0.01), and more days with loss of appetite at V4 (*p* = 0.02) and V5 (*p* < 0.001). Patients on Promelaxin experienced more days with flatulence at V5 (*p* = 0.02).

### Safety

There were 183 Treatment Emergent Adverse Events (TEAEs), defined as any event recorded by patients who took at least one IP dose (107 in the PEG and 76 in the Promelaxin arm). No TEAE was found to be causally related to the IP in each randomized arm.

### Microbiota Results

**Figure 6:**
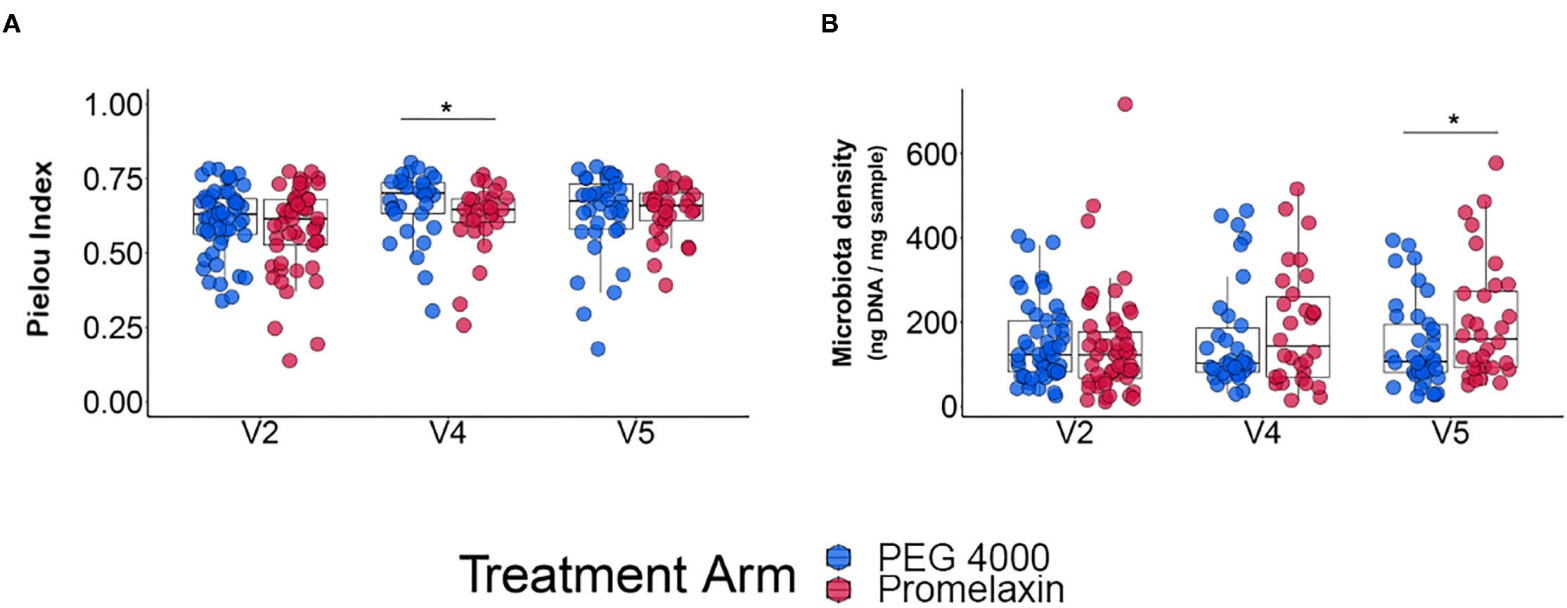
Microbiota analysis. The figure shows individual data and box-plots as median and IQR. **(A)** Microbiota evenness. **(B)** Microbiota density. Kruskal-Wallis-test and Student *T*-test were used for testing differences in microbiota evenness (Pielou's index) and microbiota density, respectively. *p < 0.05.

At V2, microbiota evenness and density were similar between the two groups (Figures 6A,B). Microbiota evenness significantly increased in the PEG 4000 arm at V4 as compared to the Promelaxin arm (*p* < 0.05; Figure 6A). In addition, at V5, patients treated with PEG showed a significantly decreased microbiota density as compared to patients treated with Promelaxin (*p* = 0.036; Figure 6B). Taxonomy data showed no major differences among the two groups of treatment (data not shown and Supplementary Material). Detailed results on microbiota indicators are reported in the Supplementary Material.

## Discussion

To the best of our knowledge, this is the first randomized, controlled, non-inferiority trial comparing a micro-enema containing Promelaxin vs. an oral osmotic laxative, i.e., PEG 4000, in 158 young children (infant and toddlers) suffering from FC. A valuable aspect of this randomized controlled trial (RCT) is the age range of the recruited population (6–48 months), since it is known that RCTs in this age range are complex (26, 27). Thus there is a considerable unmet need for treatment and a poor quality of evidence in this age range (26, 27).

Based on a standard response criterion for treatment of FC, namely the number of evacuations per week, Promelaxin microenemas were non-inferior to PEG 4000, the current reference treatment for FC (2), over a 2-week exposure. Moreover, the results of the 6-week extension of this trial (the “on-demand” phase) as well as the secondary endpoints regarding stool characteristics, were all consistent with the main result of the trial.

Since PEG is already highly effective in FC, with a reported response rate between 77 and >90% (up to 97% response rate has been reported) (28) we have run a non-inferiority trial to provide evidence that Promelaxin microenemas can be at least similarly effective and safe compared to PEG 4000, thus becoming a suitable and feasible alternative to PEG 4000. The rationale stems from reports as well as common parental experience that the oral route of drug administration, especially in toddlers and infants, is hampered by palatability of whichever formulation, and, in case of liquid formulations, also by the volume to be administered (29). Taste-related problems between 50 and 60% have also been reported for lactulose and macrogol 3550 formulations (10). Moreover, a small fraction of subjects who are treated with oral osmotic laxatives experience gastrointestinal disturbances (abdominal pain, flatulence, vomiting) which limit their usage (4). The above characteristics generate an unmet therapeutic need in a significant fraction of young children with FC, which cannot be resolved by simply changing the type of oral laxative. Finally, polymers along the gastrointestinal tract may modify the microbiota, especially upon prolonged exposure (16). Therefore, under different circumstances, equally effective and safe alternatives to PEG are needed.

FC is a functional disorder with an early onset, reported to be on average around 24 months. Moreover, early and integrated (pharmacological and non-pharmacological) interventions are needed to promptly and effectively correct FC, avoiding its persistence, complications and thus the need for chronic treatment(s). These pivotal aspects are well-reflected in the design of the present study. Indeed, the study recruited 158 children aged up to 48 months, with a median age of 24 months, thus reflecting the average age for the onset of FC, when timely interventions are crucial in interrupting the vicious loop of fecal retention (2, 6, 9, 30). In children not affected by a long-lasting constipation, a condition which is mainly attributable to defecation disorder, the use of enemas is also expected to be more effective than oral fecal softeners by virtue of a direct action on the rectum: the results of the present study are consistent with this. Moreover, the short duration of this study also reflects the importance of intervening on the functional disturbance in the shortest possible time frame, in order to rapidly resolve the symptoms and avoid unpleasant repeated experiences for the child (and possibly for the parents as well), which are a major trigger for perpetuating FC. The choice of a relatively short time for the primary analysis was supported by the finding that over a 6-day treatment course, enemas and PEG were equally effective in treating rectal fecal impaction in children (13). Moreover, it is worth noting that the trial on fecal impaction used an enema's volume of 60 ml in children aged between 4 and 6 years. Our trial used microenemas, with a far lower volume, up to 8 ml in children aged 3–4 years. Therefore, microenemas in FC may increase the feasibility and acceptability of the treatment, minimizing the unpleasant experience of the use of enemas.

Another drawback of osmotic laxatives can be their effect on the microbiota, especially upon prolonged exposure. Microbiota is particularly important in growing children, since its perturbation has been reported to potentially affect metabolic disposition in growing bodies through adulthood (31). Moreover, the gut microbiota of children seems to be much less resilient than that of adults (32, 33). A reduced density of microbiota has been reported in mice exposed to PEG (14). We observed at V5 a reduced density of microbiota in PEG vs. Promelaxin, albeit the relative reduction was small (Figure 6), which may be consistent with experimental animal data. On the other hand, the Pielou's index was decreased at V4 in the Promelaxin group, indicating a reduction of evenness index of alpha diversity. Although these data may suggest a potential lower impact of microenemas on the microbiota as compared to PEG 4000, they remain hypothesis-generating and await further studies to understand their clinical relevance.

Thus, our study shows that microenemas with Promelaxin, a completely biodegradable substance system, being non-inferior to PEG 4000 in efficacy and with no major concerns on safety, are a valuable alternative to PEG, especially for those children for whom PEG administration can be problematic, i.e., in very young children with low compliance or low acceptance of an oral formulation due to poor palatability. An additional value of microenema treatment option is suggested by the present data comparing the on-demand use and the response rates throughout the observational period. The lower on-demand use of microenemas occurred in the context of comparable response for stool frequency and consistency, suggesting that Promelaxin may somehow help restore the physiological bowel function.

Despite the fact that non-pharmacological strategies are relevant for FC (2), head-to-head comparisons with the standard of care so far have been too limited (5) to provide sound evidence that can guide physicians and inform guidelines. A head-to-head comparison of enemas and PEG has only been performed with regard to fecal impaction (13), a disorder that is related, though different, to FC and that requires a fast intervention for a prompt resolution (4). Our randomized head-to-head comparison of a pharmacological vs. a non-pharmacological product (a medical device made of substances) in 158 young children can provide sound evidence-based, as well as useful, information to address an unmet therapeutic need. Thus, Promelaxin can offer a personalized treatment of FC in young children.

Our study does have some limitations. First, this is a relatively short-term study, thus we can only hypothesize that this treatment may be also useful in chronic FC when longer exposure is needed, even though the “on-demand” phase lasted for 6 weeks and the overall duration was 8 weeks. Moreover, the results of the “on demand” phase, where Promelaxin was used for 33% fewer days are also indicative of a possible positive effect in the long term. Importantly, it is key to quickly interrupt the vicious loop of FC in order to avoid prolonging the disorder and making it chronic. Another limitation is the age range of the patients (up to 48 months), which means that we can only infer similar results beyond this age range. However, it should be emphasized that the age range of this study closely reflects the age for the onset of FC. Moreover, in this age range oral drug administration can be more problematic as compared to older children, thus likely reflecting an unmet need in infants and toddlers.

In conclusions, Promelaxin microenemas have a similar efficacy, tolerability and safety compared to oral PEG, thus representing a valuable option for the treatment of FC in young children, and they may also help to individualize and optimize the treatment of FC in the early stages.

## Data Availability Statement

The datasets presented in this article are not readily available because of the following reason related to the application of European data protection laws (specifically GDPR): meta-genomic data-files should be deposit in a public repository, whose country code Top-Level Domain is UK, that is abroad the EEA. Since the unique identifier of the interested person is included, these data do not qualify as anonymous data, but as (pseudonymized) personal data, and, for this reason, they are not transferable to the requested repository, that is outside the European Union, due to the limitations indicated in the text of the informed consent provided to enrolled subjects, which specifies that Personal data will not be transferred abroad. Requests to access the datasets should be directed to the Promoter of the study (who owns data property), at the following e-mail address: ClinicalTrials@aboca.it.

## Ethics Statement

The study protocol was approved by the local ethical committees of each participating center (coordinating center approval date: 09/09/2015, approval number 190/15). The written informed consent to participation in the study was obtained from the parents of all patients prior to their enrollment.

## Author Contributions

CS, VC, and MR: conception and design, acquisition, analysis and interpretation of data, drafting the article, and final approval of the version to be published. CT, GM, AV, SC, CC, VD'A, LS, and AS: conception and design, acquisition, drafting the article, and final approval of the version to be published. All authors contributed to the article and approved the submitted version.

## Funding

This study was sponsored by Aboca, the manufacturer of the medical device (i.e., the test product; brand name: Melilax Pediatric). The sponsor was not involved in the collection, analysis, interpretation of data, the writing of this article, or the decision to submit it for publication.

## Conflict of Interest

AS is clinical investigator for Janssen Biologics B.V. and consultant for Angelini; she was clinical investigator for Aboca and PAREXEL International Srl; she was consultant for Aboca, for D.M.G. Italy and Nestlé, she was data safety monitoring board member for Sucampo AG and speaker for Aboca, Angelini, D.M.G. Italy and Valeas. The remaining authors declare that the research was conducted in the absence of any commercial or financial relationships that could be construed as a potential conflict of interest.

## Publisher's Note

All claims expressed in this article are solely those of the authors and do not necessarily represent those of their affiliated organizations, or those of the publisher, the editors and the reviewers. Any product that may be evaluated in this article, or claim that may be made by its manufacturer, is not guaranteed or endorsed by the publisher.
