# Supplementary material for: Promelaxin Microenemas Are Non-inferior to Oral Polyethylene Glycol for the Treatment of Functional Constipation in Young Children: A Randomized Clinical Trial
Source: Front Pediatr. 2021 Oct 29;9:753938. doi: 10.3389/fped.2021.753938 (PMC8586088; doi:10.3389/fped.2021.753938)
Supplement: Supplementary file 1 [file Presentation_1.PPTX]

## Slide 1
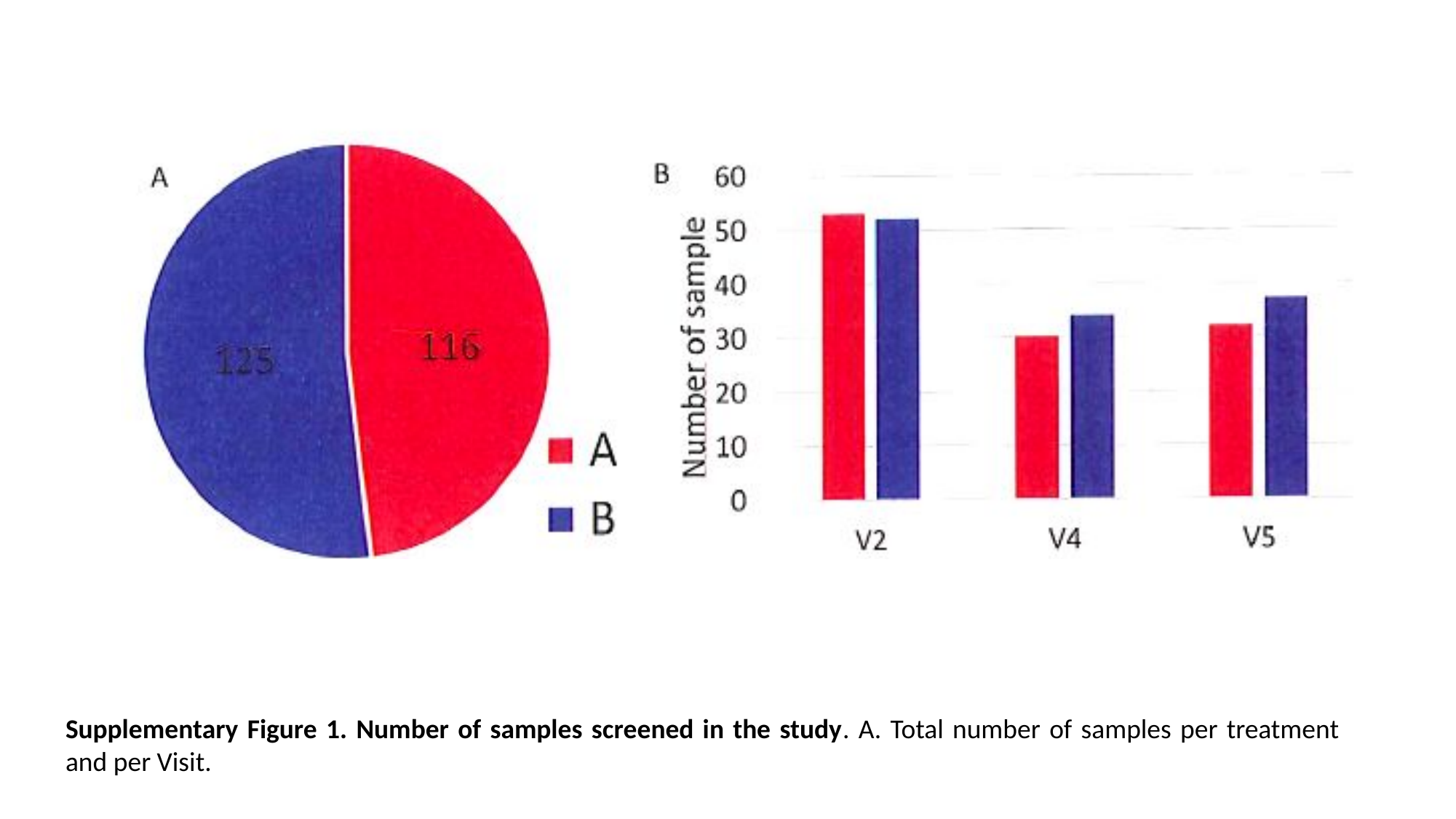

Supplementary Figure 1. Number of samples screened in the study. A. Total number of samples per treatment and per Visit.

## Slide 2
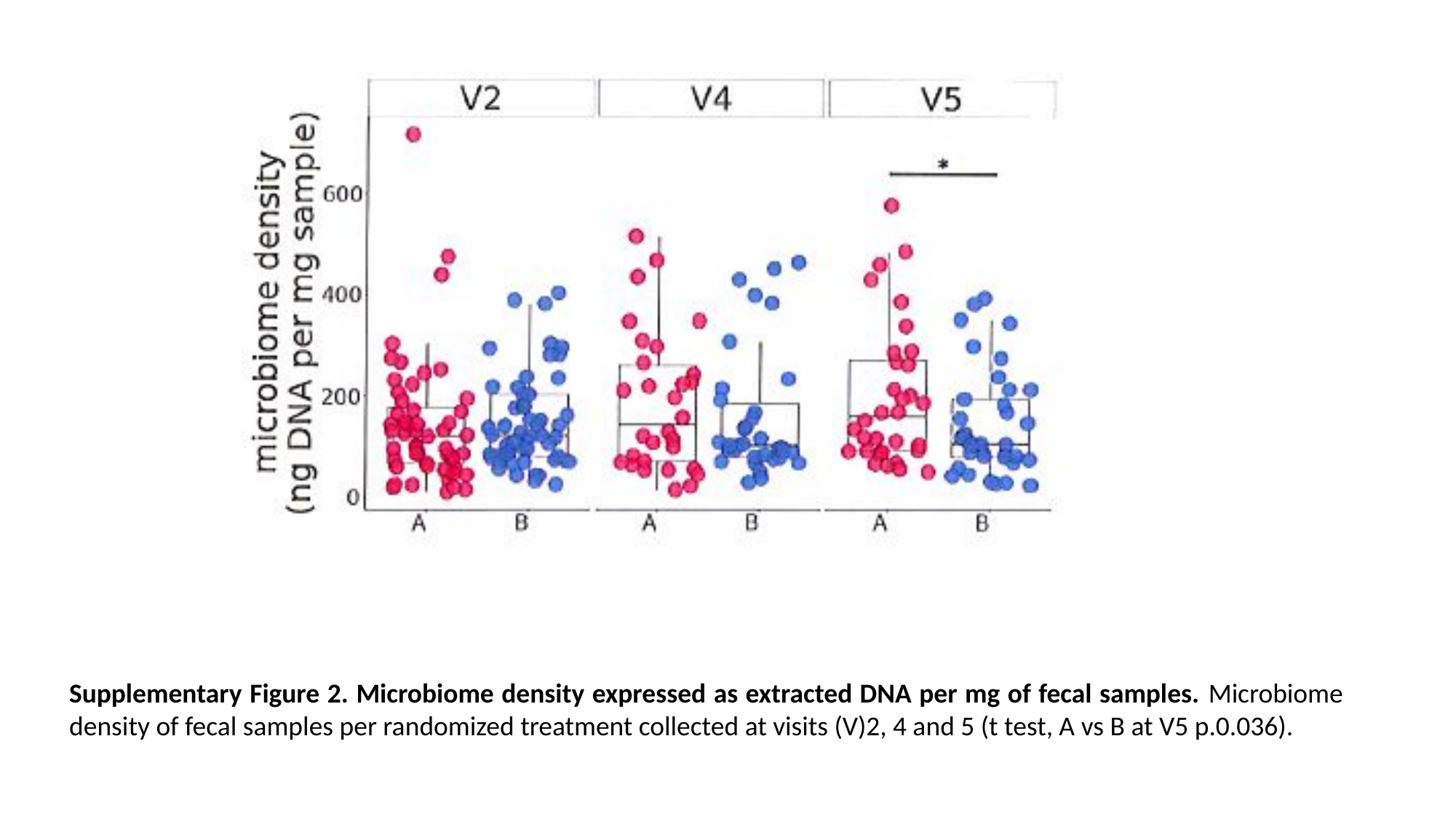

Supplementary Figure 2. Microbiome density expressed as extracted DNA per mg of fecal samples. Microbiome density of fecal samples per randomized treatment collected at visits (V)2, 4 and 5 (t test, A vs B at V5 p.0.036).

## Slide 3
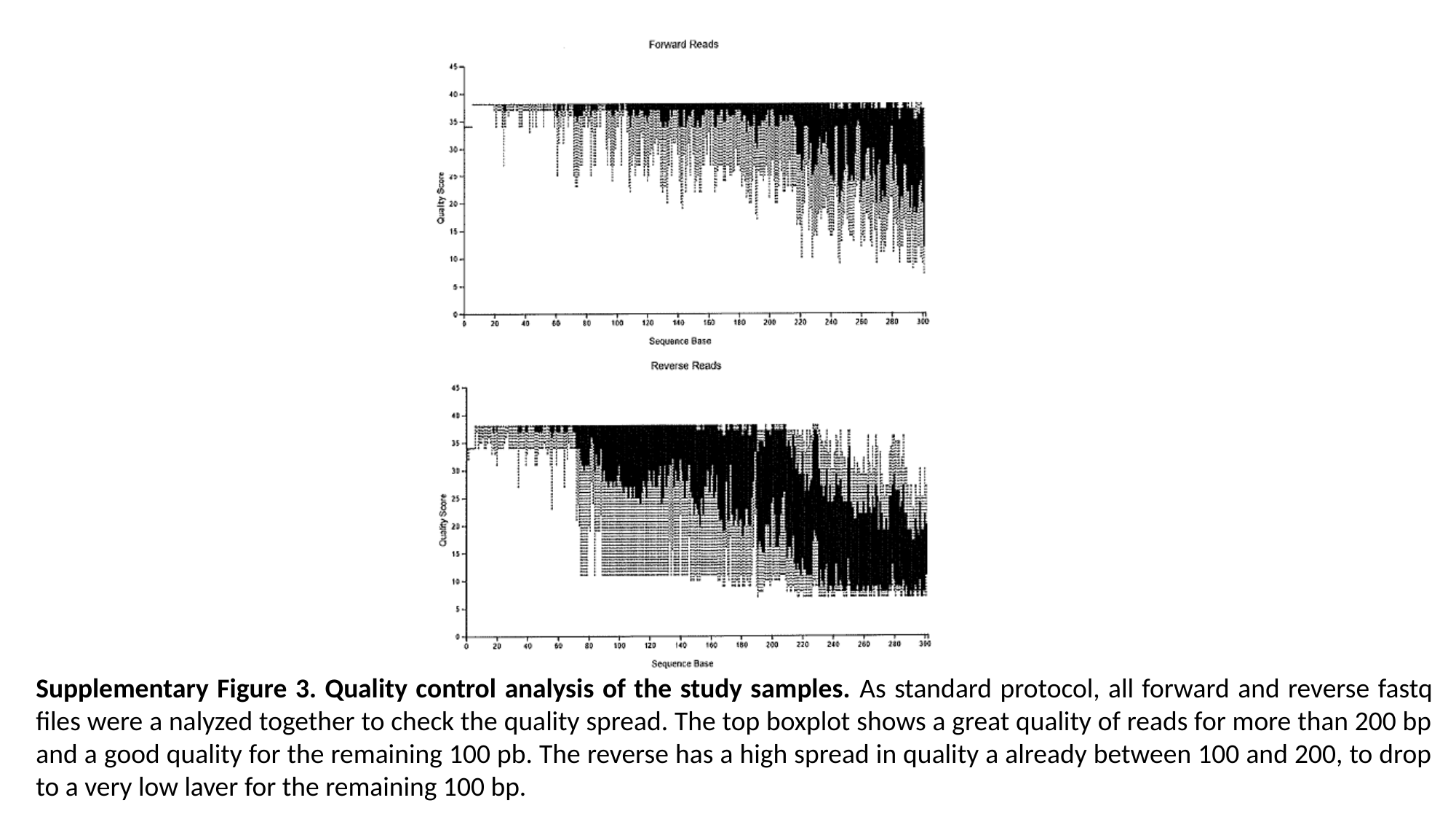

Supplementary Figure 3. Quality control analysis of the study samples. As standard protocol, all forward and reverse fastq files were a nalyzed together to check the quality spread. The top boxplot shows a great quality of reads for more than 200 bp and a good quality for the remaining 100 pb. The reverse has a high spread in quality a already between 100 and 200, to drop to a very low laver for the remaining 100 bp.

## Slide 4
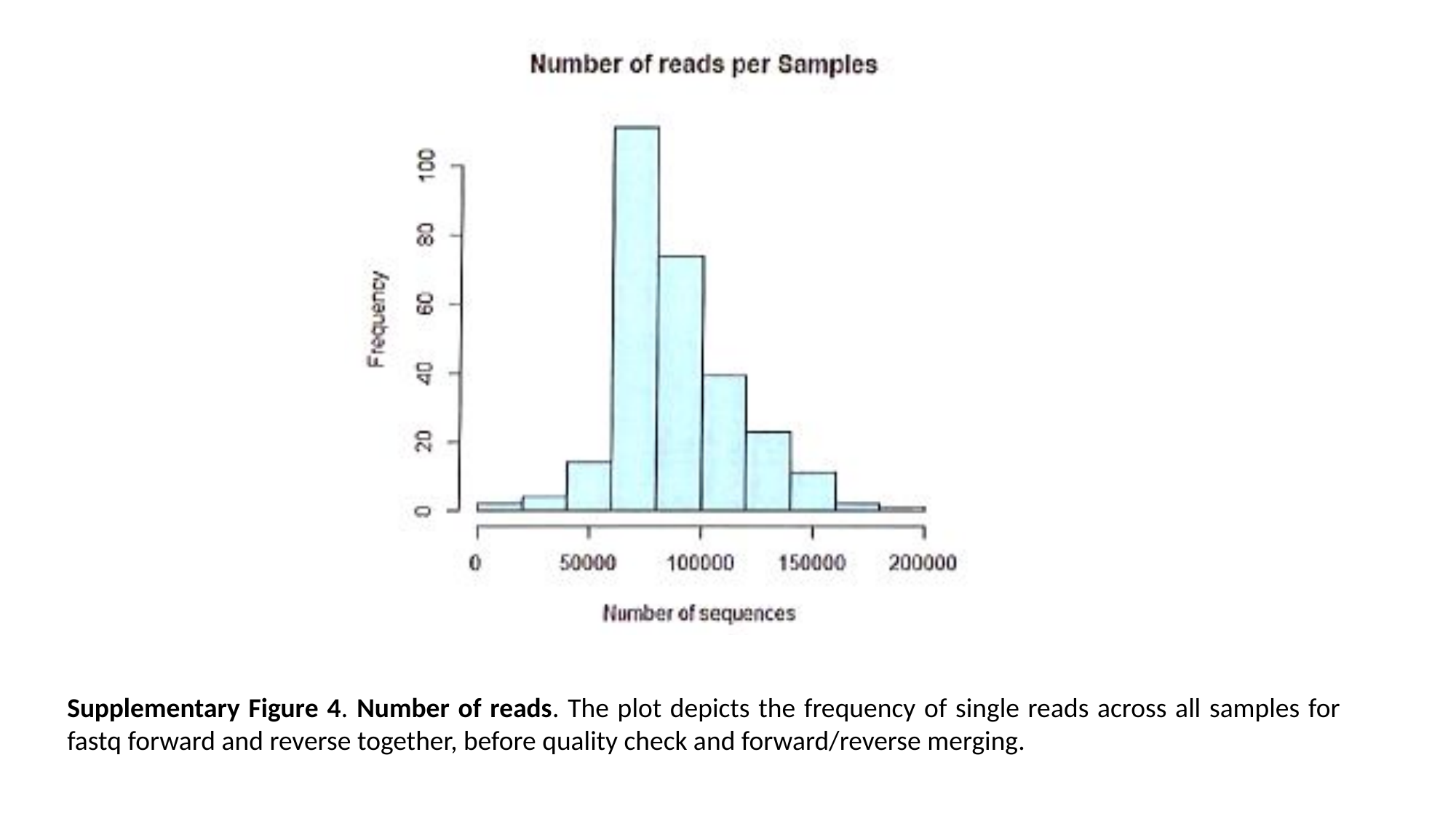

Supplementary Figure 4. Number of reads. The plot depicts the frequency of single reads across all samples for fastq forward and reverse together, before quality check and forward/reverse merging.

## Slide 5
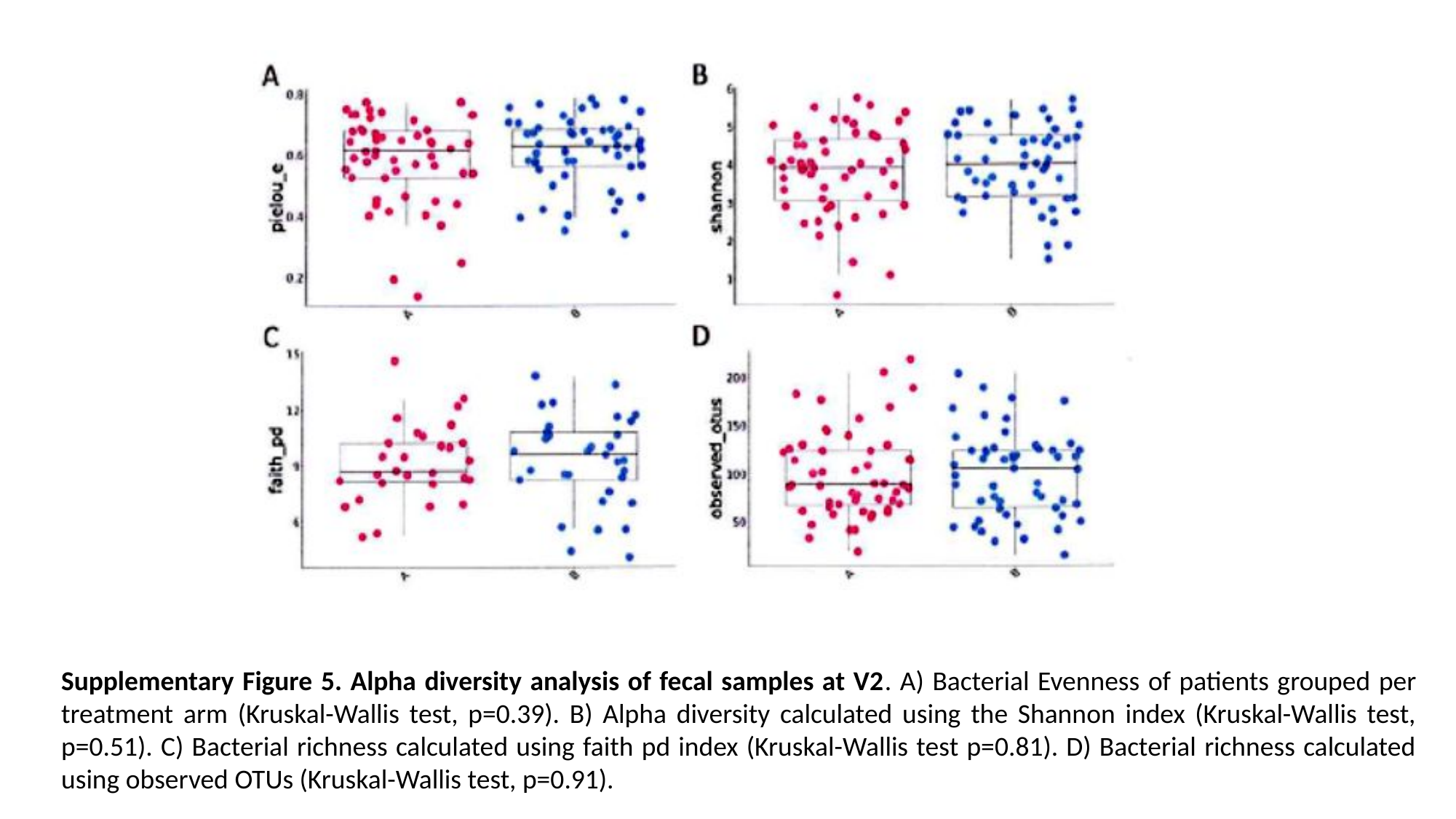

Supplementary Figure 5. Alpha diversity analysis of fecal samples at V2. A) Bacterial Evenness of patients grouped per treatment arm (Kruskal-Wallis test, p=0.39). B) Alpha diversity calculated using the Shannon index (Kruskal-Wallis test, p=0.51). C) Bacterial richness calculated using faith pd index (Kruskal-Wallis test p=0.81). D) Bacterial richness calculated using observed OTUs (Kruskal-Wallis test, p=0.91).

## Slide 6
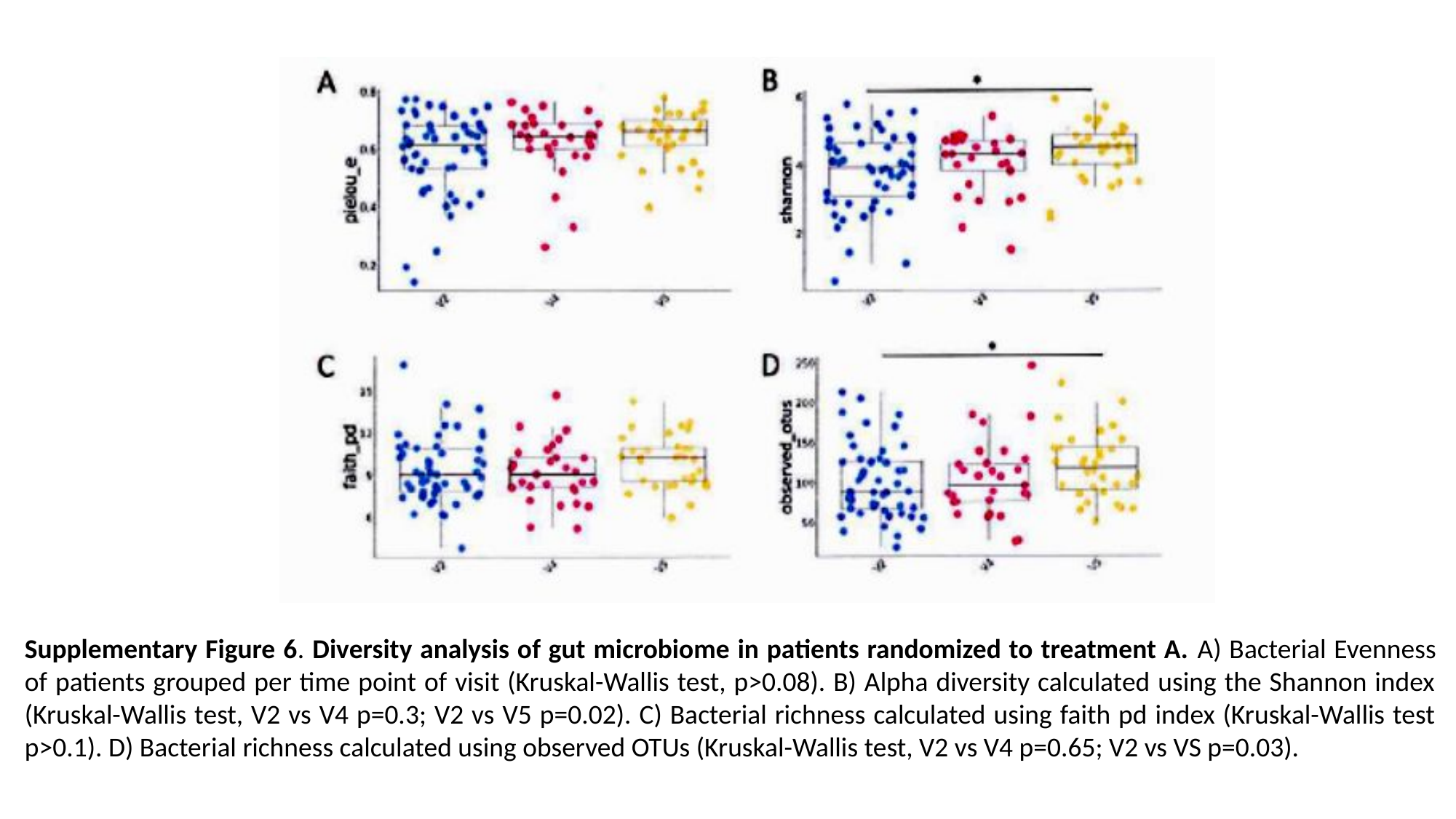

Supplementary Figure 6. Diversity analysis of gut microbiome in patients randomized to treatment A. A) Bacterial Evenness of patients grouped per time point of visit (Kruskal-Wallis test, p>0.08). B) Alpha diversity calculated using the Shannon index (Kruskal-Wallis test, V2 vs V4 p=0.3; V2 vs V5 p=0.02). C) Bacterial richness calculated using faith pd index (Kruskal-Wallis test p>0.1). D) Bacterial richness calculated using observed OTUs (Kruskal-Wallis test, V2 vs V4 p=0.65; V2 vs VS p=0.03).

## Slide 7
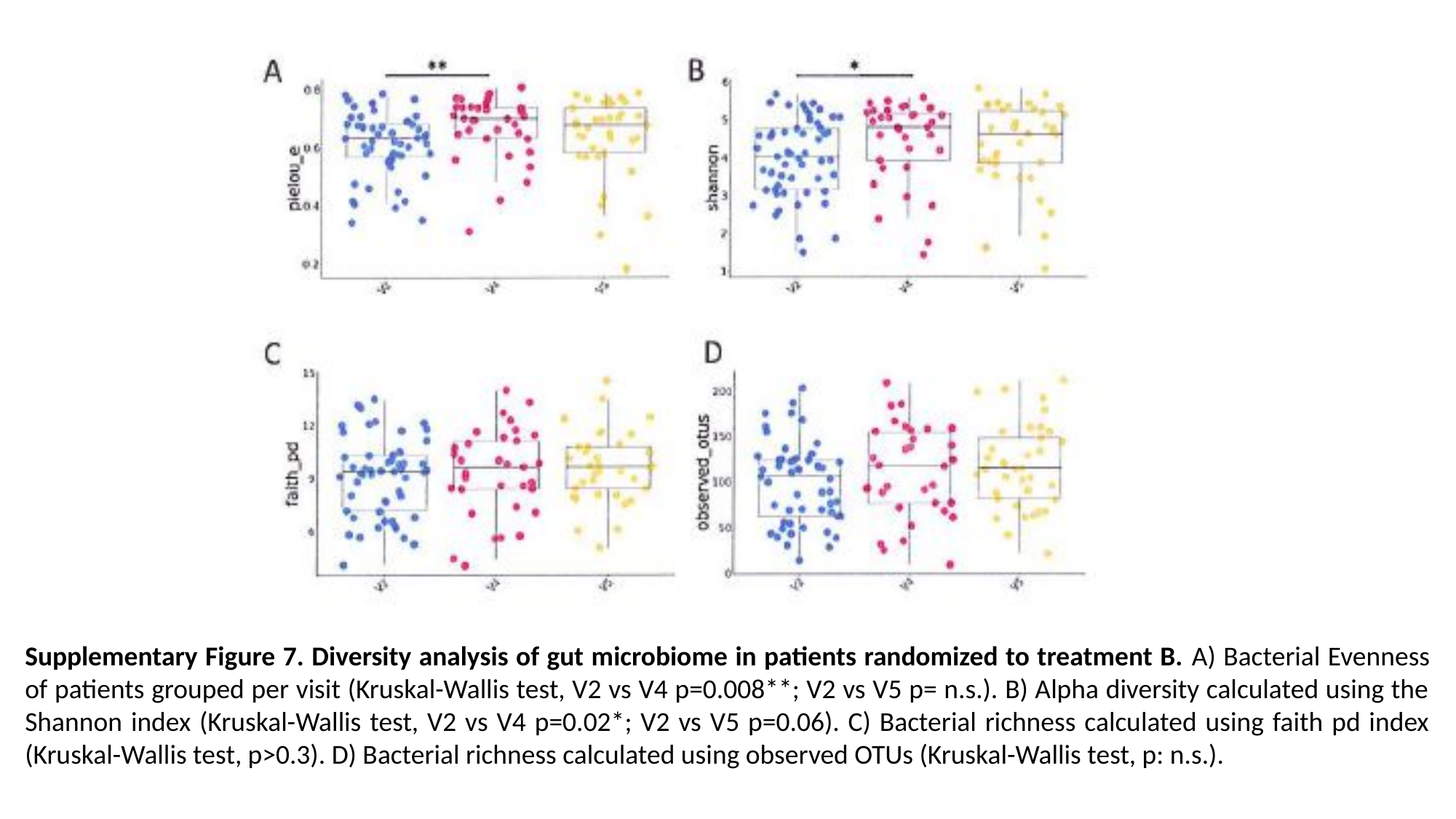

Supplementary Figure 7. Diversity analysis of gut microbiome in patients randomized to treatment B. A) Bacterial Evenness of patients grouped per visit (Kruskal-Wallis test, V2 vs V4 p=0.008**; V2 vs V5 p= n.s.). B) Alpha diversity calculated using the Shannon index (Kruskal-Wallis test, V2 vs V4 p=0.02*; V2 vs V5 p=0.06). C) Bacterial richness calculated using faith pd index (Kruskal-Wallis test, p>0.3). D) Bacterial richness calculated using observed OTUs (Kruskal-Wallis test, p: n.s.).

## Slide 8
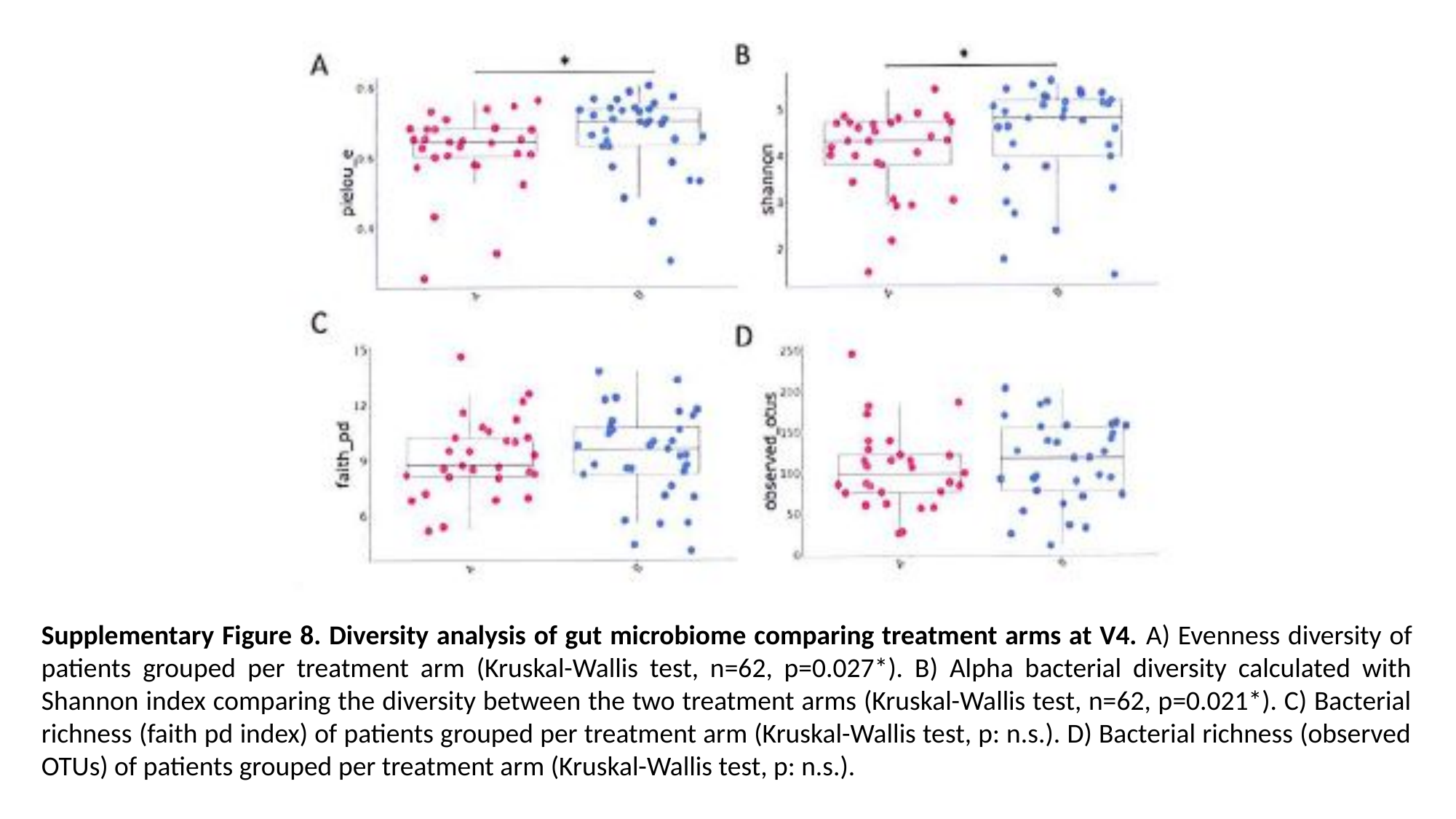

Supplementary Figure 8. Diversity analysis of gut microbiome comparing treatment arms at V4. A) Evenness diversity of patients grouped per treatment arm (Kruskal-Wallis test, n=62, p=0.027*). B) Alpha bacterial diversity calculated with Shannon index comparing the diversity between the two treatment arms (Kruskal-Wallis test, n=62, p=0.021*). C) Bacterial richness (faith pd index) of patients grouped per treatment arm (Kruskal-Wallis test, p: n.s.). D) Bacterial richness (observed OTUs) of patients grouped per treatment arm (Kruskal-Wallis test, p: n.s.).

## Slide 9
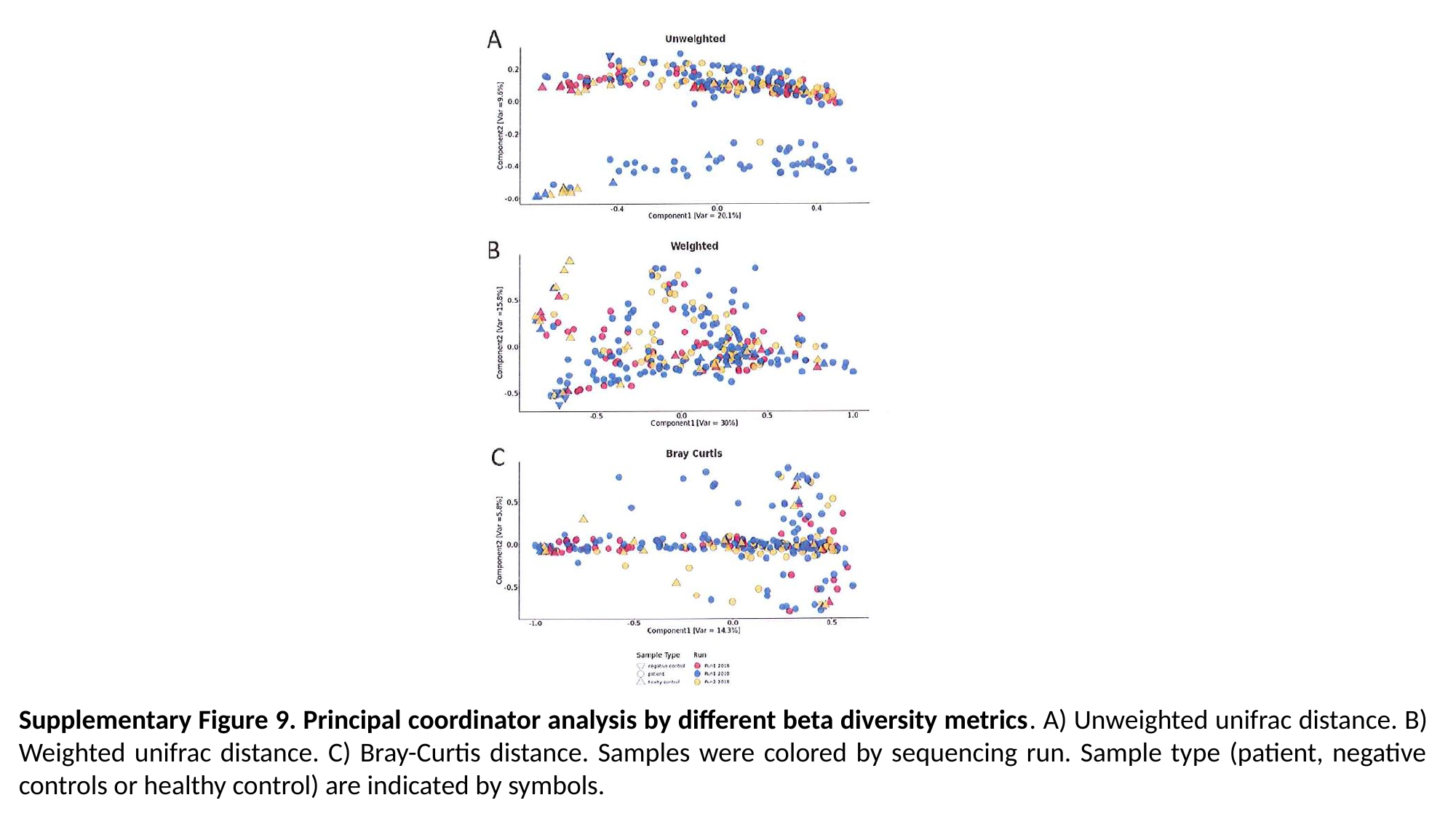

Supplementary Figure 9. Principal coordinator analysis by different beta diversity metrics. A) Unweighted unifrac distance. B) Weighted unifrac distance. C) Bray-Curtis distance. Samples were colored by sequencing run. Sample type (patient, negative controls or healthy control) are indicated by symbols.

## Slide 10
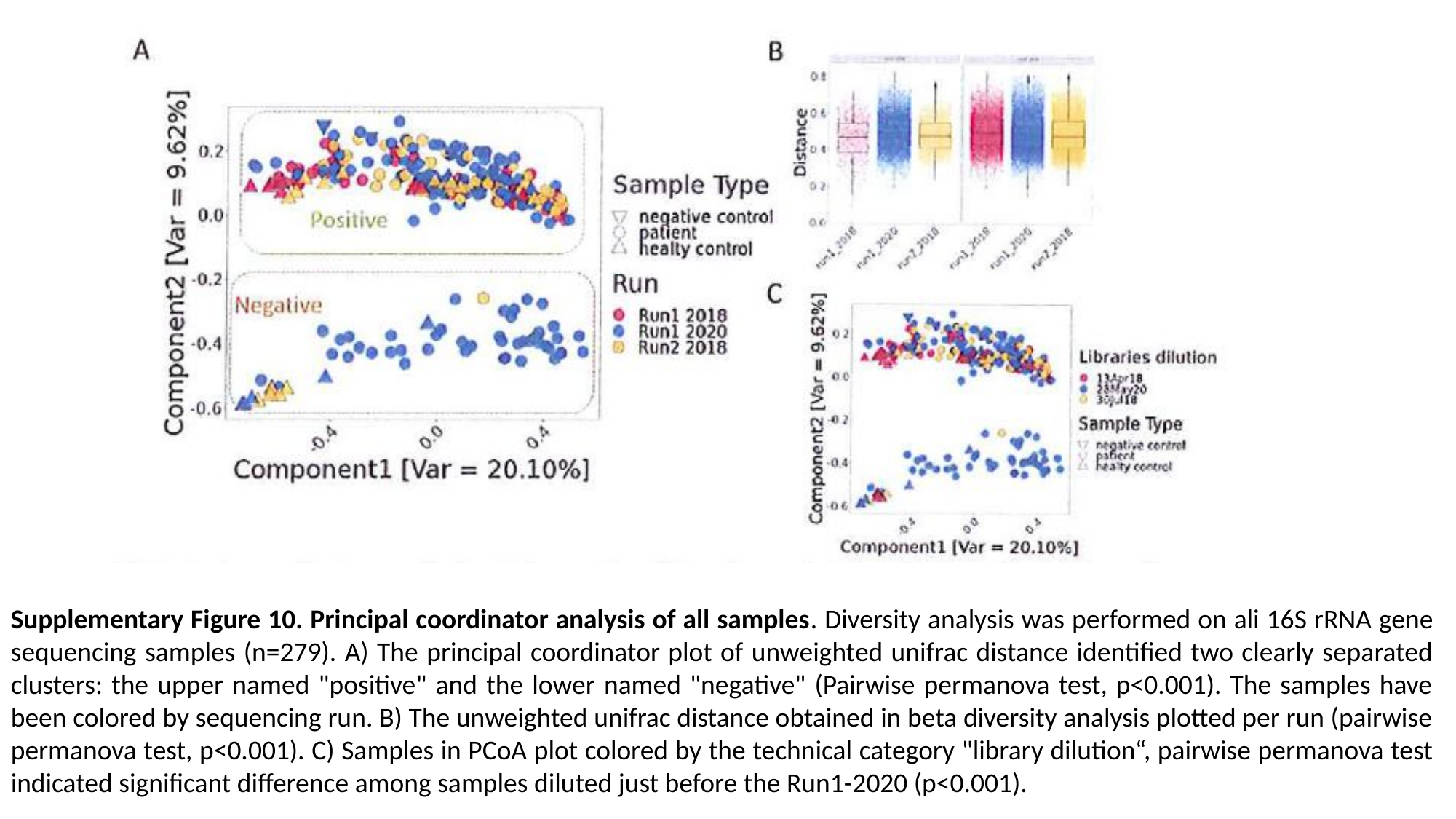

Supplementary Figure 10. Principal coordinator analysis of all samples. Diversity analysis was performed on ali 16S rRNA gene sequencing samples (n=279). A) The principal coordinator plot of unweighted unifrac distance identified two clearly separated clusters: the upper named "positive" and the lower named "negative" (Pairwise permanova test, p<0.001). The samples have been colored by sequencing run. B) The unweighted unifrac distance obtained in beta diversity analysis plotted per run (pairwise permanova test, p<0.001). C) Samples in PCoA plot colored by the technical category "library dilution“, pairwise permanova test indicated significant difference among samples diluted just before the Run1-2020 (p<0.001).

## Slide 11
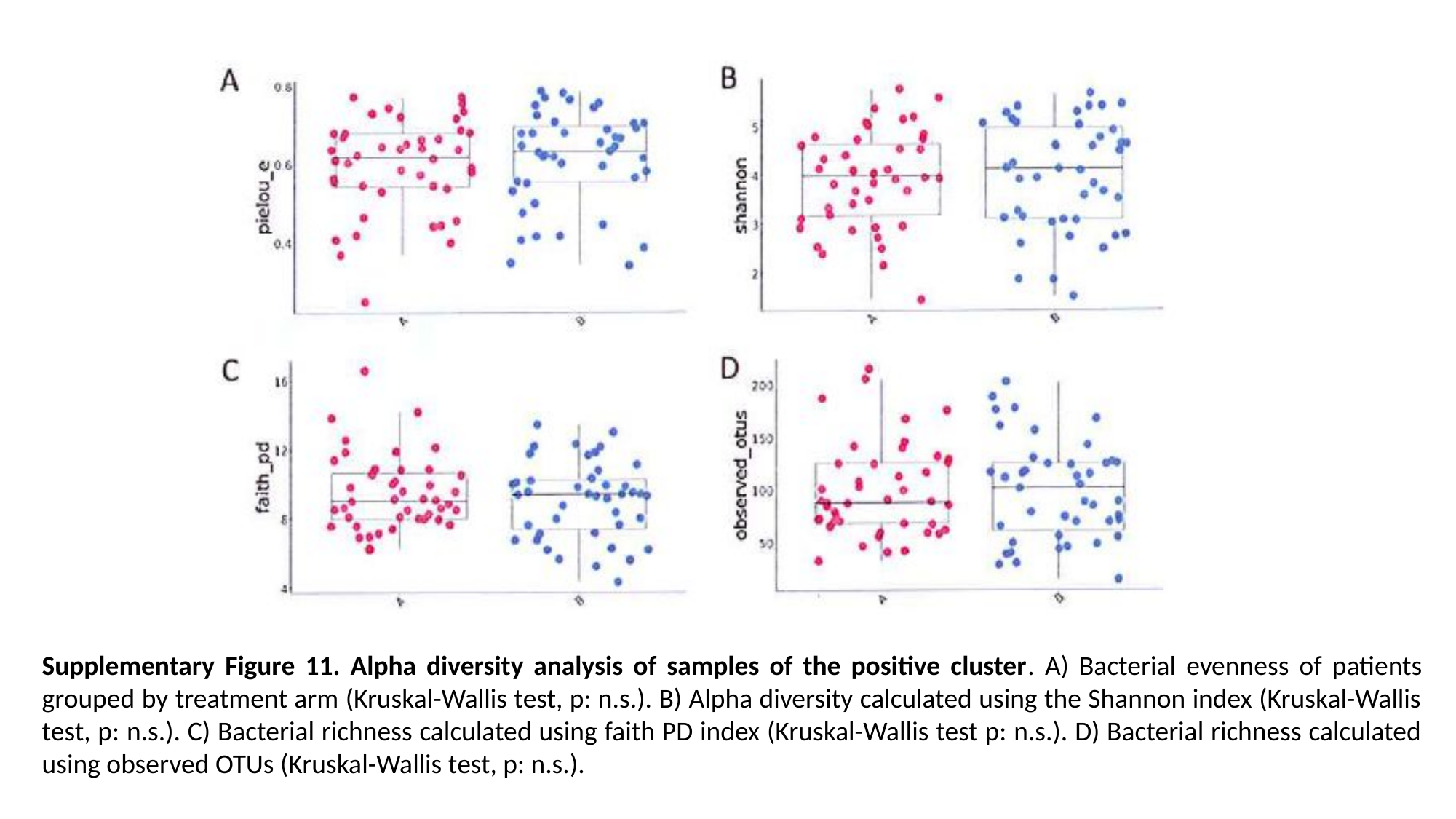

Supplementary Figure 11. Alpha diversity analysis of samples of the positive cluster. A) Bacterial evenness of patients grouped by treatment arm (Kruskal-Wallis test, p: n.s.). B) Alpha diversity calculated using the Shannon index (Kruskal-Wallis test, p: n.s.). C) Bacterial richness calculated using faith PD index (Kruskal-Wallis test p: n.s.). D) Bacterial richness calculated using observed OTUs (Kruskal-Wallis test, p: n.s.).

## Slide 12
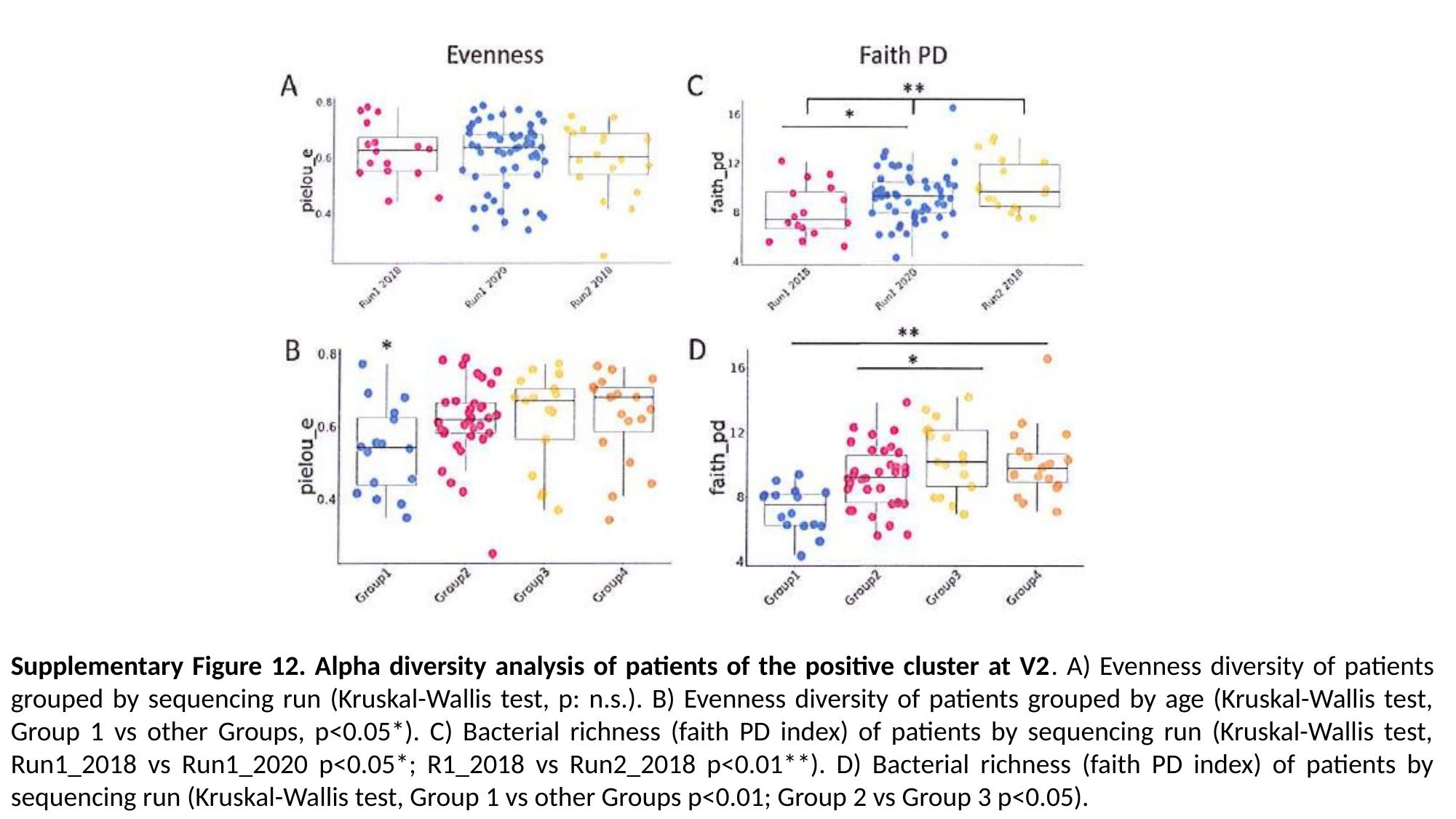

Supplementary Figure 12. Alpha diversity analysis of patients of the positive cluster at V2. A) Evenness diversity of patients grouped by sequencing run (Kruskal-Wallis test, p: n.s.). B) Evenness diversity of patients grouped by age (Kruskal-Wallis test, Group 1 vs other Groups, p<0.05*). C) Bacterial richness (faith PD index) of patients by sequencing run (Kruskal-Wallis test, Run1_2018 vs Run1_2020 p<0.05*; R1_2018 vs Run2_2018 p<0.01**). D) Bacterial richness (faith PD index) of patients by sequencing run (Kruskal-Wallis test, Group 1 vs other Groups p<0.01; Group 2 vs Group 3 p<0.05).

## Slide 13
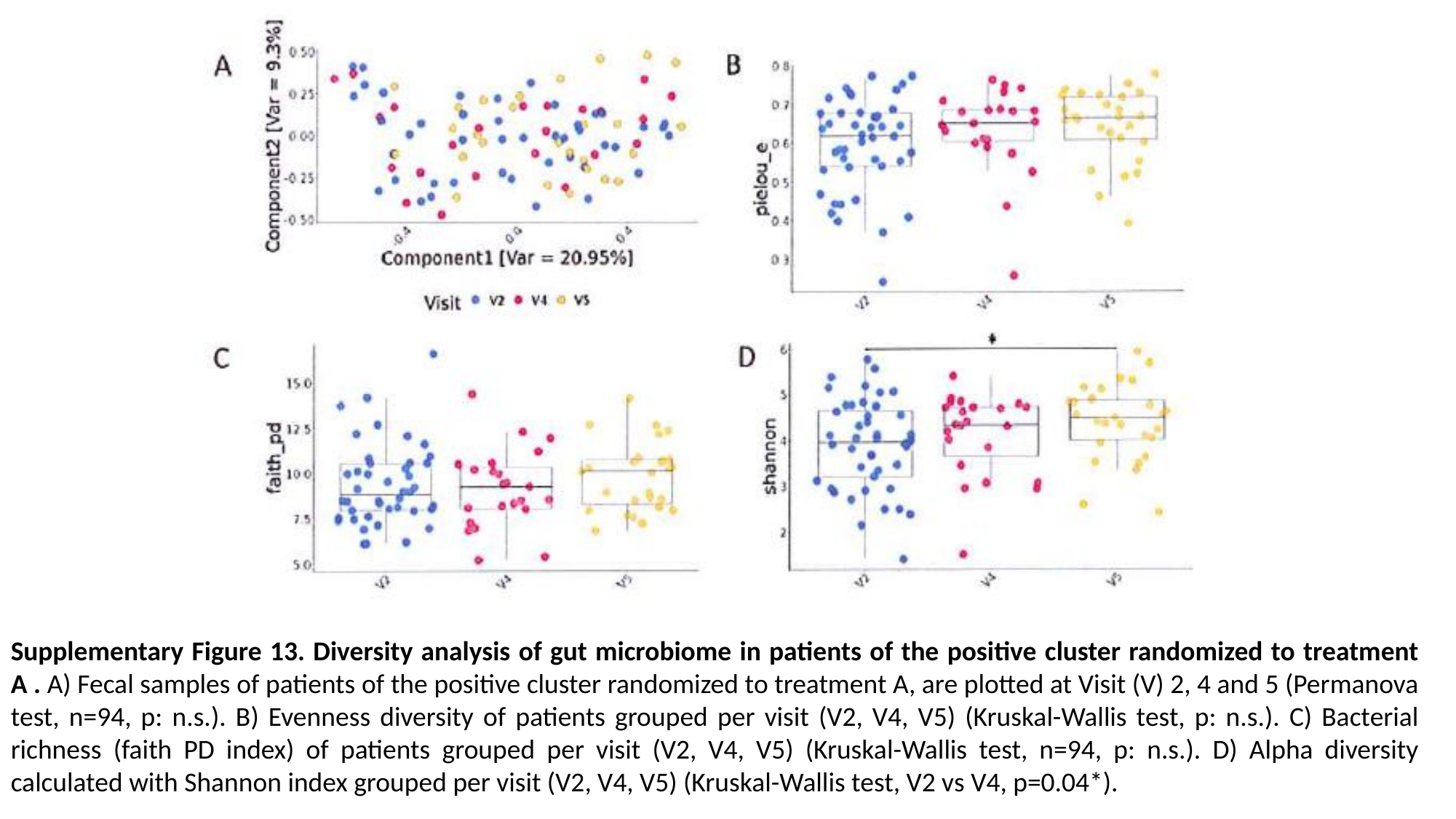

Supplementary Figure 13. Diversity analysis of gut microbiome in patients of the positive cluster randomized to treatment A . A) Fecal samples of patients of the positive cluster randomized to treatment A, are plotted at Visit (V) 2, 4 and 5 (Permanova test, n=94, p: n.s.). B) Evenness diversity of patients grouped per visit (V2, V4, V5) (Kruskal-Wallis test, p: n.s.). C) Bacterial richness (faith PD index) of patients grouped per visit (V2, V4, V5) (Kruskal-Wallis test, n=94, p: n.s.). D) Alpha diversity calculated with Shannon index grouped per visit (V2, V4, V5) (Kruskal-Wallis test, V2 vs V4, p=0.04*).

## Slide 14
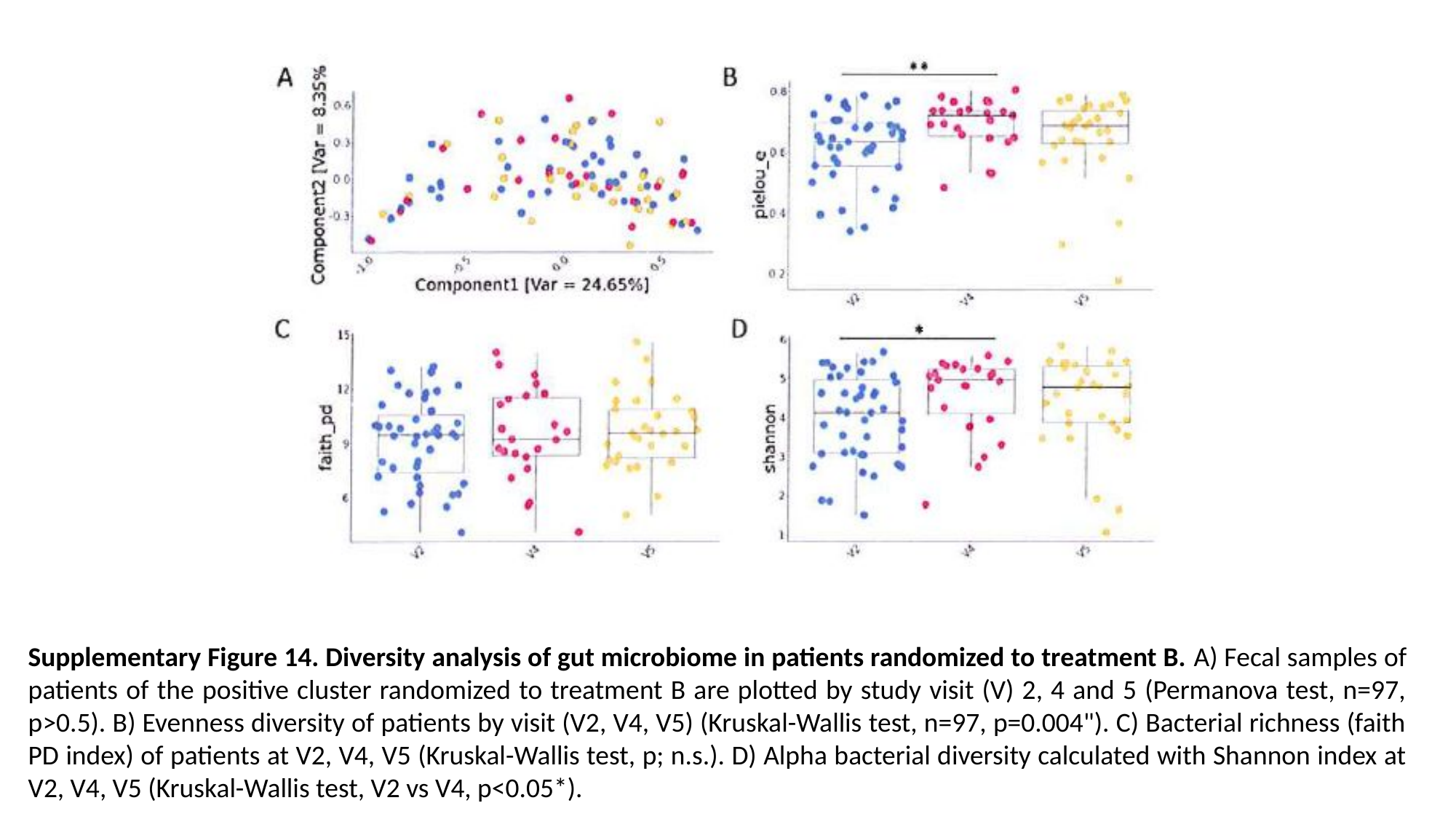

Supplementary Figure 14. Diversity analysis of gut microbiome in patients randomized to treatment B. A) Fecal samples of patients of the positive cluster randomized to treatment B are plotted by study visit (V) 2, 4 and 5 (Permanova test, n=97, p>0.5). B) Evenness diversity of patients by visit (V2, V4, V5) (Kruskal-Wallis test, n=97, p=0.004"). C) Bacterial richness (faith PD index) of patients at V2, V4, V5 (Kruskal-Wallis test, p; n.s.). D) Alpha bacterial diversity calculated with Shannon index at V2, V4, V5 (Kruskal-Wallis test, V2 vs V4, p<0.05*).

## Slide 15
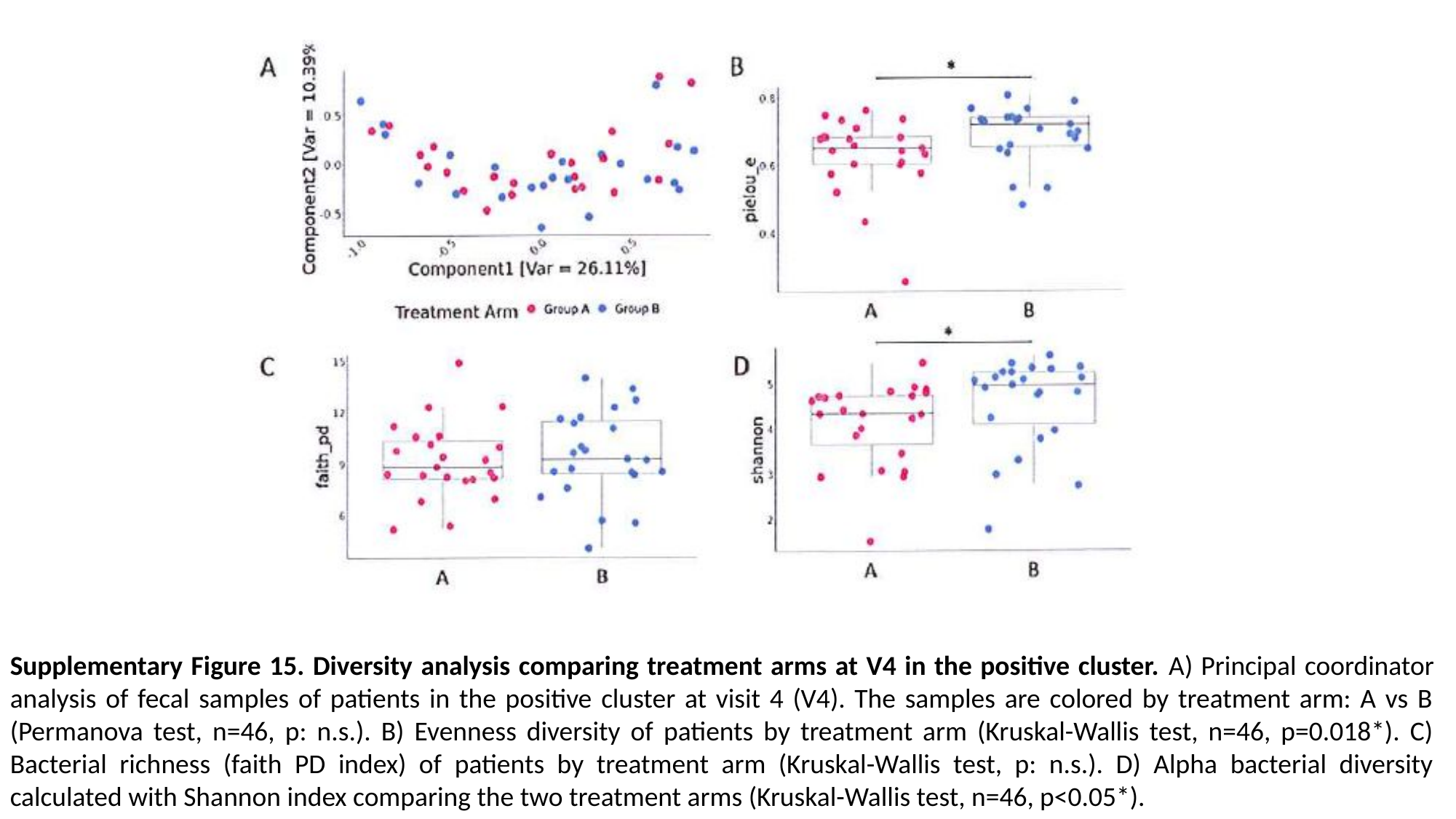

Supplementary Figure 15. Diversity analysis comparing treatment arms at V4 in the positive cluster. A) Principal coordinator analysis of fecal samples of patients in the positive cluster at visit 4 (V4). The samples are colored by treatment arm: A vs B (Permanova test, n=46, p: n.s.). B) Evenness diversity of patients by treatment arm (Kruskal-Wallis test, n=46, p=0.018*). C) Bacterial richness (faith PD index) of patients by treatment arm (Kruskal-Wallis test, p: n.s.). D) Alpha bacterial diversity calculated with Shannon index comparing the two treatment arms (Kruskal-Wallis test, n=46, p<0.05*).

## Slide 16
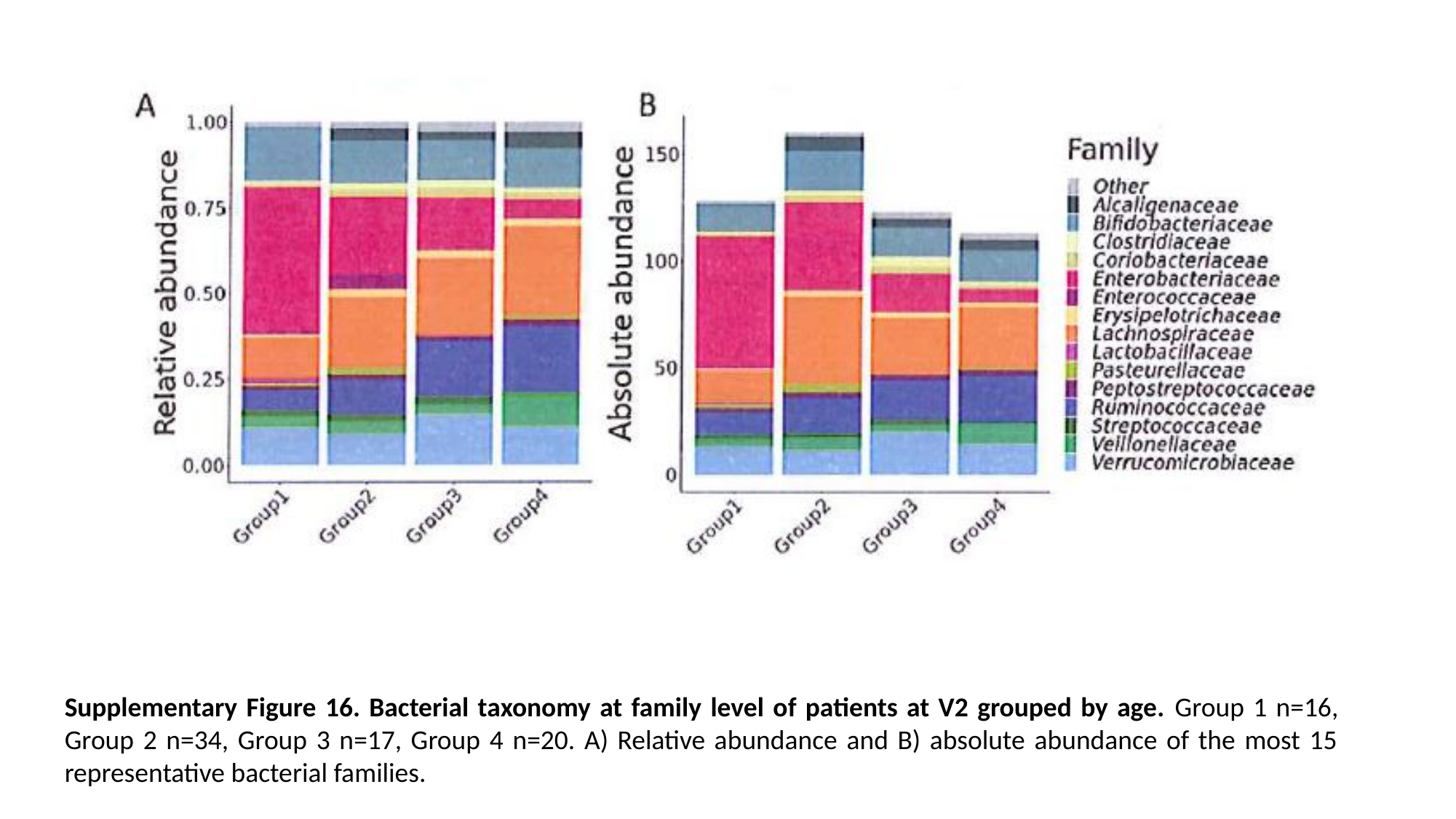

Supplementary Figure 16. Bacterial taxonomy at family level of patients at V2 grouped by age. Group 1 n=16, Group 2 n=34, Group 3 n=17, Group 4 n=20. A) Relative abundance and B) absolute abundance of the most 15 representative bacterial families.

## Slide 17
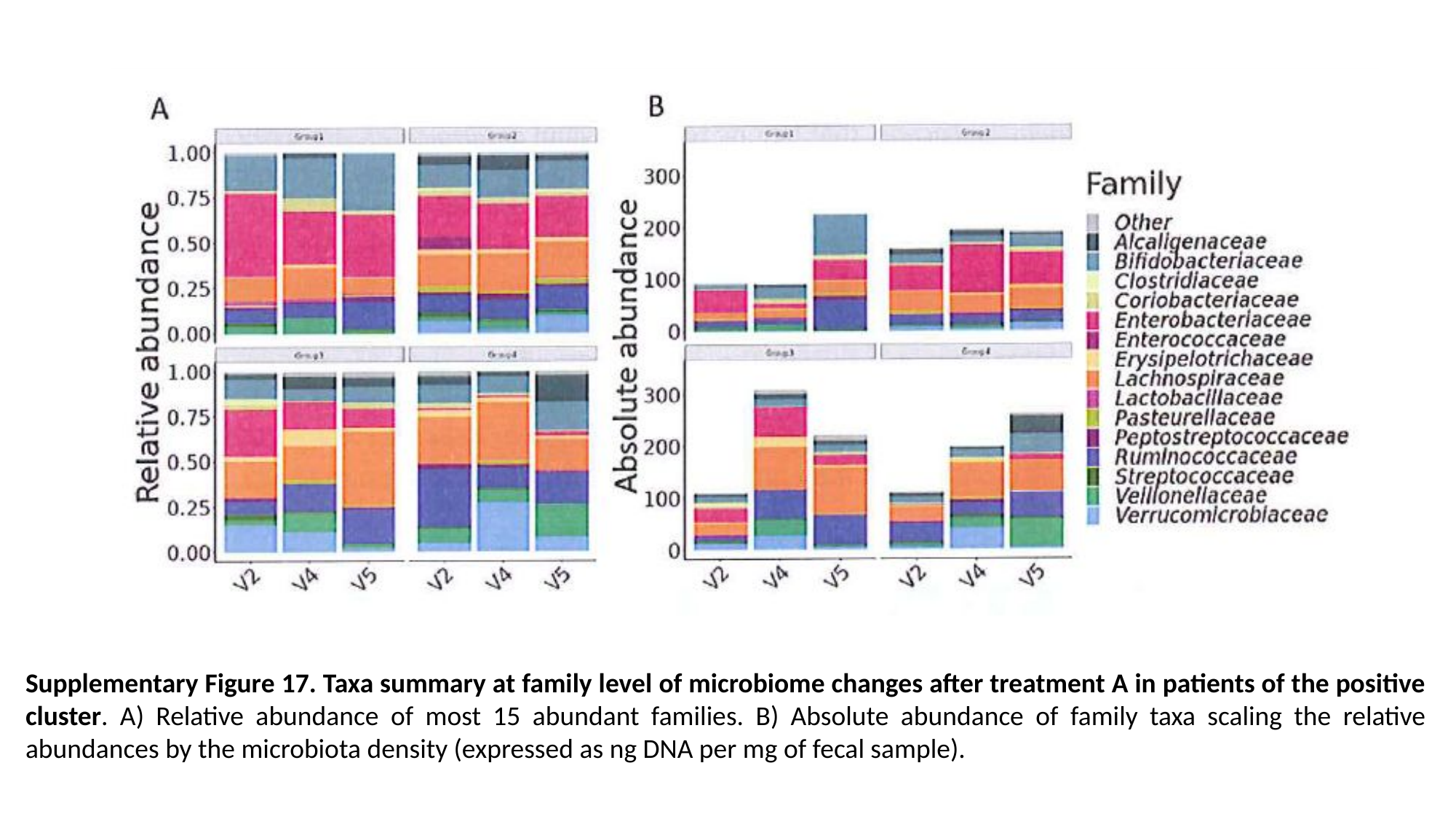

Supplementary Figure 17. Taxa summary at family level of microbiome changes after treatment A in patients of the positive cluster. A) Relative abundance of most 15 abundant families. B) Absolute abundance of family taxa scaling the relative abundances by the microbiota density (expressed as ng DNA per mg of fecal sample).

## Slide 18
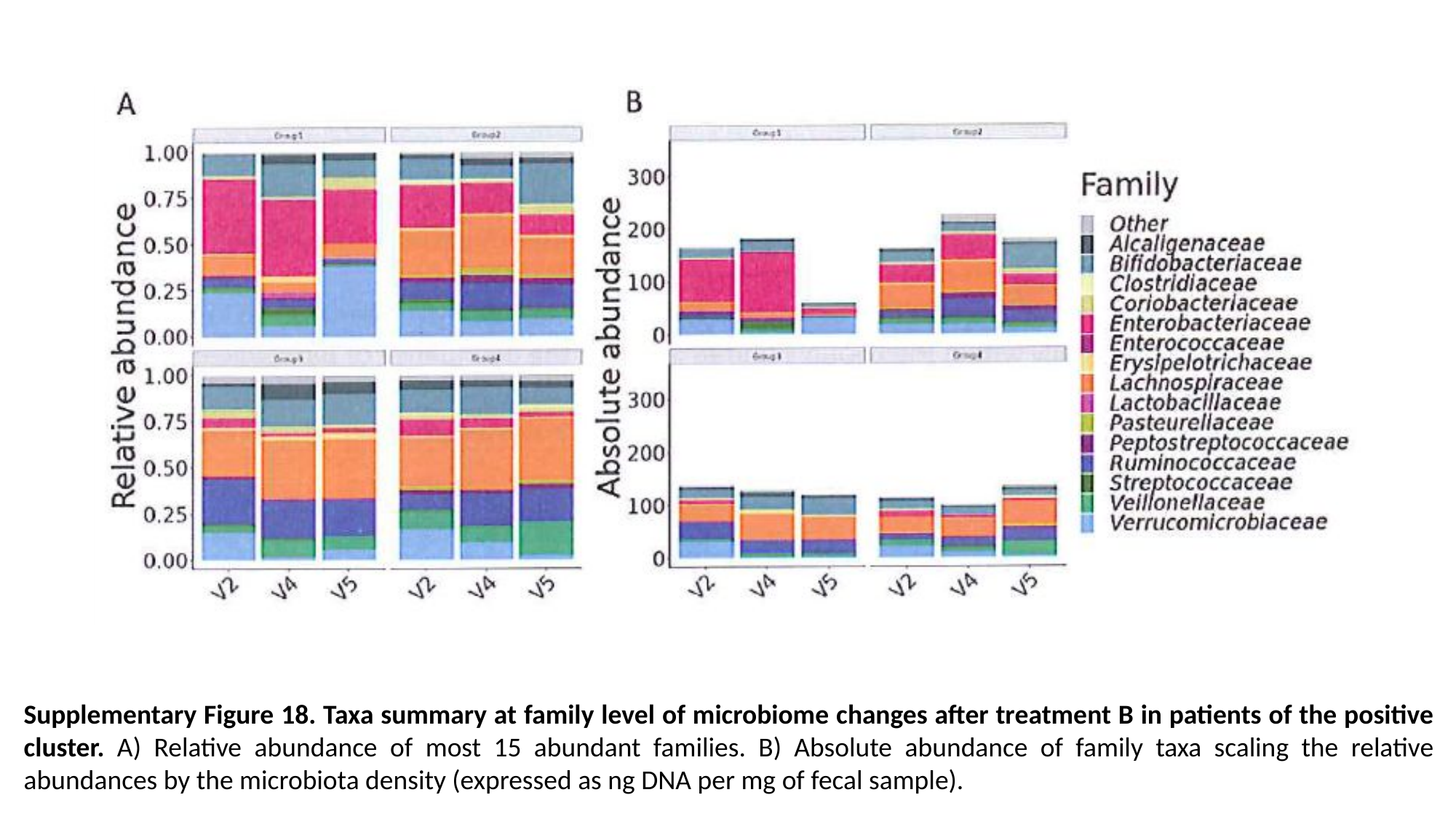

Supplementary Figure 18. Taxa summary at family level of microbiome changes after treatment B in patients of the positive cluster. A) Relative abundance of most 15 abundant families. B) Absolute abundance of family taxa scaling the relative abundances by the microbiota density (expressed as ng DNA per mg of fecal sample).

## Slide 19
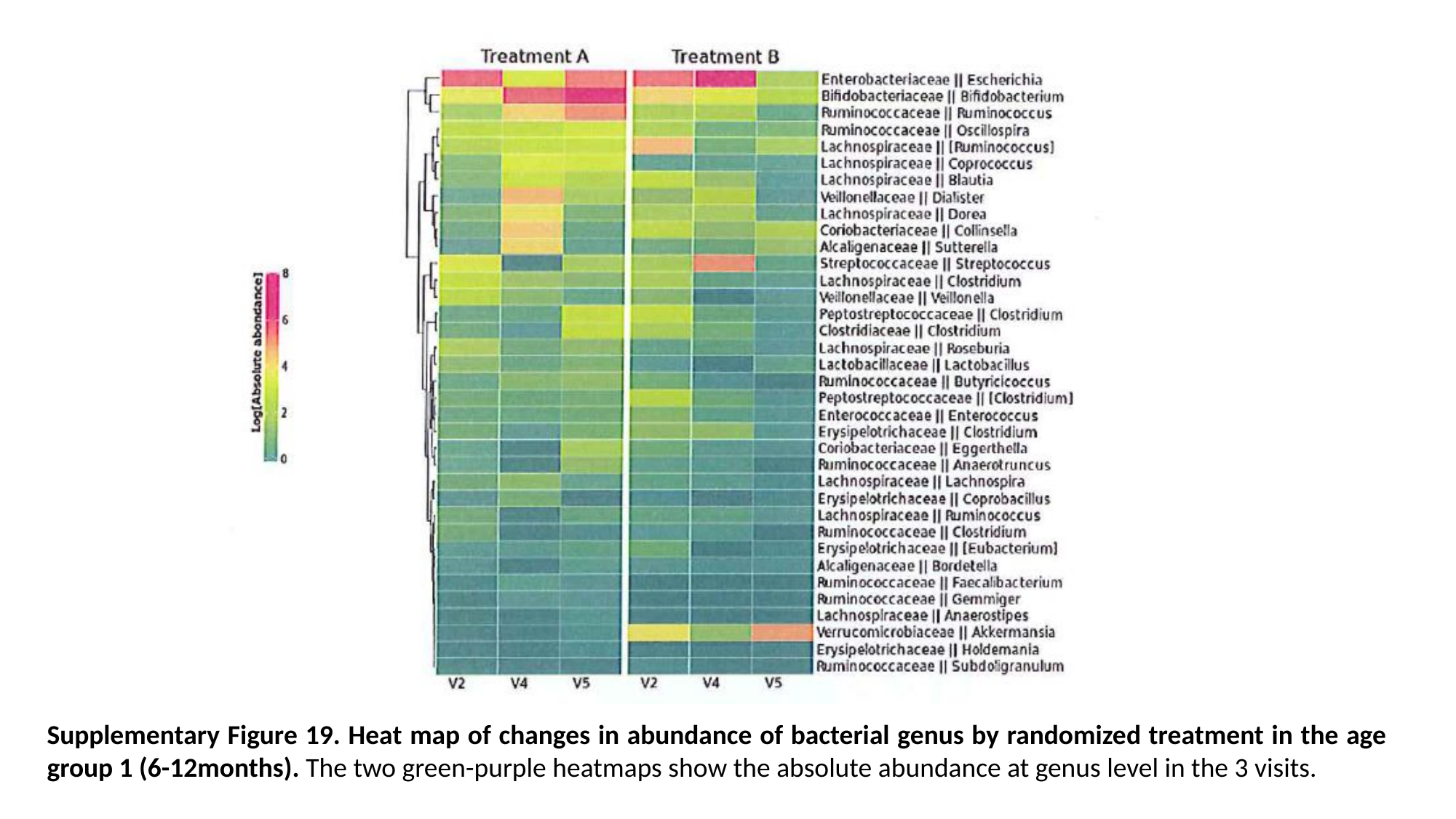

Supplementary Figure 19. Heat map of changes in abundance of bacterial genus by randomized treatment in the age group 1 (6-12months). The two green-purple heatmaps show the absolute abundance at genus level in the 3 visits.

## Slide 20
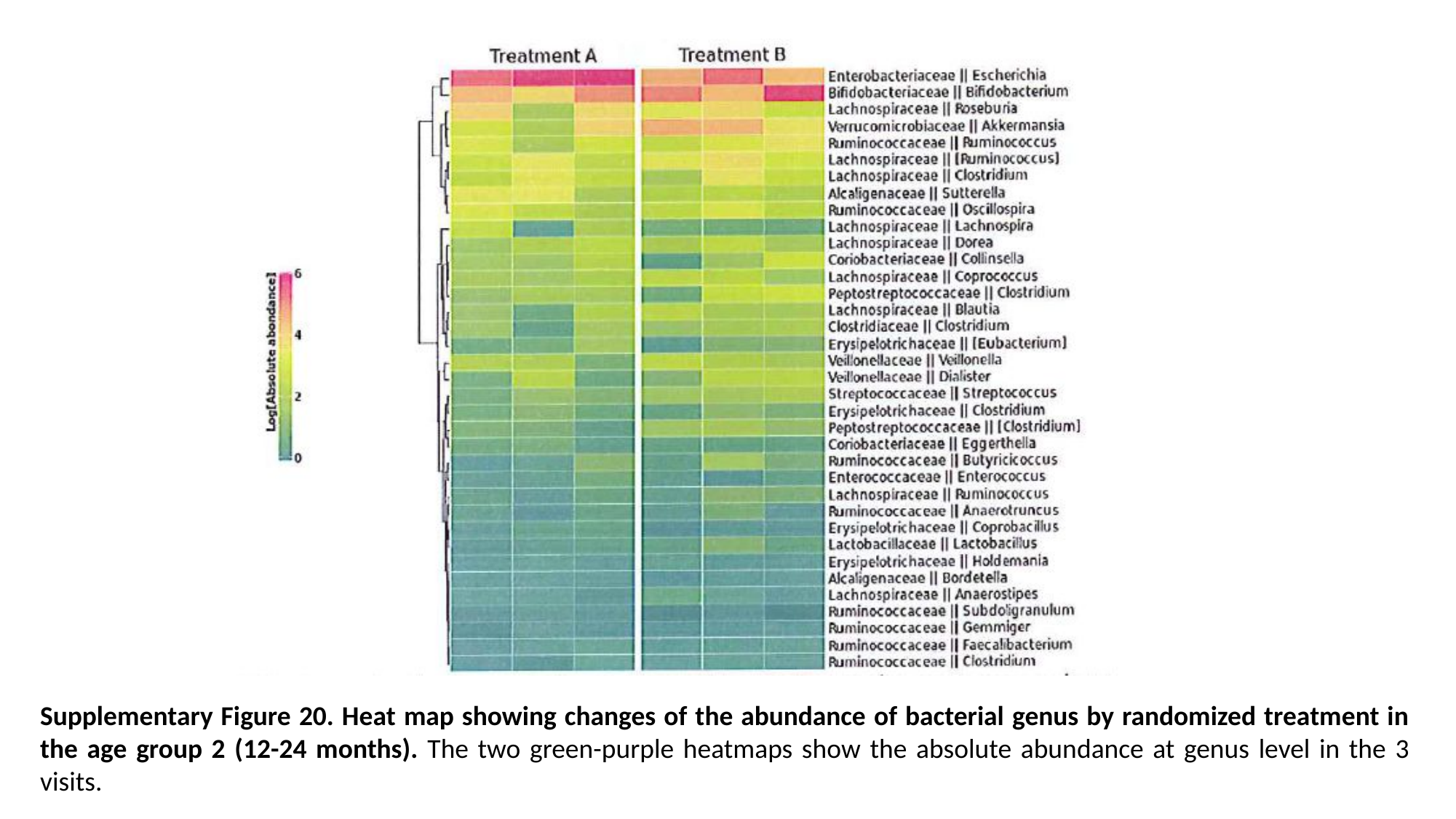

Supplementary Figure 20. Heat map showing changes of the abundance of bacterial genus by randomized treatment in the age group 2 (12-24 months). The two green-purple heatmaps show the absolute abundance at genus level in the 3 visits.

## Slide 21
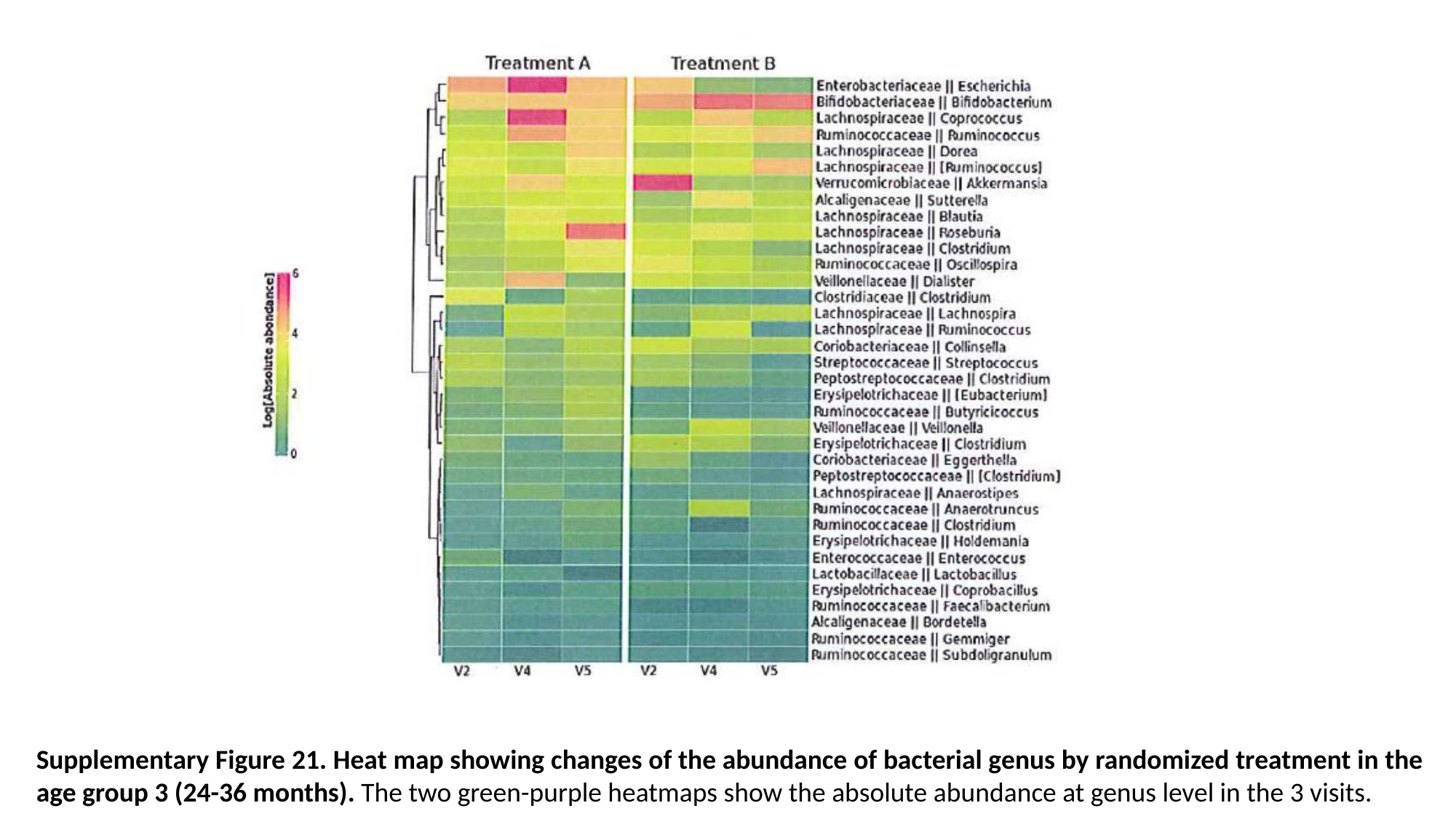

Supplementary Figure 21. Heat map showing changes of the abundance of bacterial genus by randomized treatment in the age group 3 (24-36 months). The two green-purple heatmaps show the absolute abundance at genus level in the 3 visits.

## Slide 22
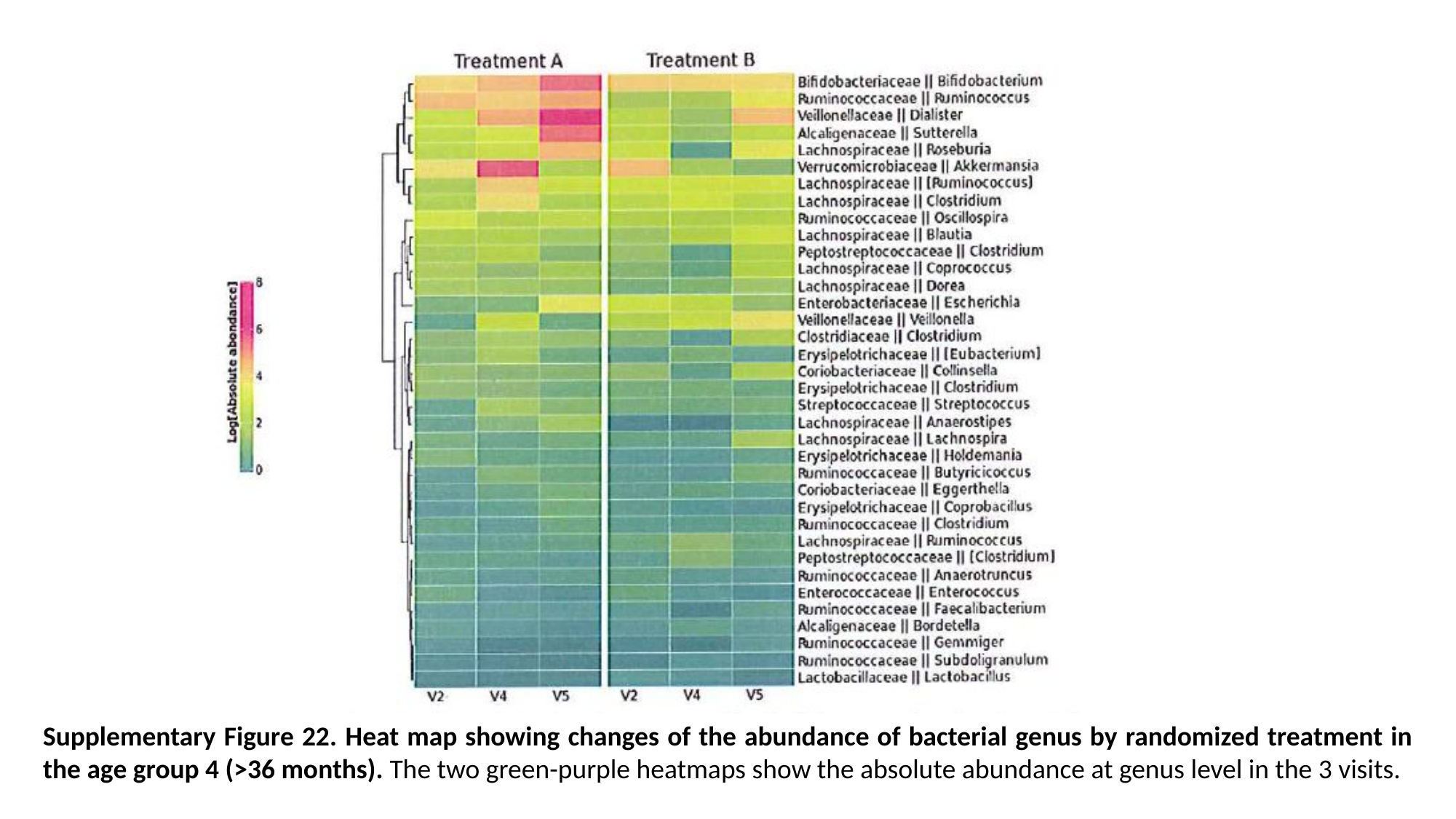

Supplementary Figure 22. Heat map showing changes of the abundance of bacterial genus by randomized treatment in the age group 4 (>36 months). The two green-purple heatmaps show the absolute abundance at genus level in the 3 visits.
