# Supplementary material for: Promelaxin Microenemas Are Non-inferior to Oral Polyethylene Glycol for the Treatment of Functional Constipation in Young Children: A Randomized Clinical Trial
Source: Front Pediatr. 2021 Oct 29;9:753938. doi: 10.3389/fped.2021.753938 (PMC8586088; doi:10.3389/fped.2021.753938)
Supplement: Supplementary file 2 [file Data_Sheet_1.docx]

Promelaxin microenemas are non-inferior to oral Polyethylene Glycol for the Treatment of Functional Constipation in Young Children: a randomized clinical trial

**Supplementary material content 1**

**Microbiome analysis**

*Sample description*

The initial number of patients recruited for this study was 106: among these patients, 64 hadthe sample at V4 and 69 at V5, resulting in 64 patients presenting all the 3 timepoints, the remaining patients had 1 or 2 timepoints. In total 239 fecal samples were collected. Patients in the 2 Treatment Arms (A and B; Promelaxin and PEG) had approximately the same size along all the study as reported in Supplementary figure 1.

*Microbiome density*

Microbiota density, ie is the amount of bacteria per microgram of fecal sample expressed as ng of DNA per mg of fecal sampleof patients grouped by treatment arm and visits (Supplementary figure 2). The comparison showed no differences between the patients randomized in the two treatments at the baseline (V2). At visit V4 we observed a higher microbiome density on average for the patients under treatment A as compared to patients under treatment B, and at V5 we have a significant higher microbiome density in patient treated with A than B (Supplementary figure 2, t-test, p=0.036).

*Quality control of sequencing analysis*

A total of 281 samples were sequenced: 36 were positive controls, 6 negative controls and 239 collected from patients.

Samples were processed and sequenced in 3 separate runs (2 in 2018 -May and June — and 1 in 2020 — September). Sequencing was performed using MiSeq Illumina sequencing 2x300 platform in paired end, using amplification primers for the region V4 V6 for 16S rRNA gene (-600 bp length) for bacterial microbiome. After Iast sequencing run, fastq files were demultiplexed and trimmed to remove Illumina adapters, and two fastq files (forward and reverse) for each sample have been generated. Sequencing results were analyzed using the software Qiime2, by a standard pipeline. Paired end sequencing with MiSeq 2x300 for a region of "600 bp does not permit an overlap between forward and reverse reads during analysis and generate low quality of flanking region for longer reads.

*Alpha diversity analysis*

Alpha diversity analysis summarizes the structure of an ecological community with respect to its richness (number of taxonomic groups) or evenness (distribution of abundances of the groups). The diversity results depend on the diversity indexes that have been used.

**Alpha diversity at baseline** (V2). We performed alpha diversity analyses in all patients at baseline (before randomized treatment, V2). Alpha diversity analyses of patient's samples showed non significant differences between the patients randomized to the two treatment arms: treatment A n=52 and treatment B n=53 (Supplementary figure 5, n=105, p n.s.).

**Alpha diversity during treatment A.** We performed diversity microbiome analysis on fecal samples of patients randomized to treatment A (n=113), in order to assess whether the treatment A could induce changes in gut microbiome diversity and composition. Kruskal-Wallis test showed non significant differences in bacterial evenness among the time points (Supplementary figure 6A, p n.s.). Richness analyses based on Faith PD diversity index also showed no difference along the treatment time points, whilst diversity analyses using the Shannon index and observed OTUs index identified a higher bacterial diversity in gut microbiome of patients at V5 versus V2 (Supplementary figure 6B-D, p=0.02 and p=0.03, respectively).

**Alpha diversity during treatment B.** We performed diversity microbiome analysis on fecal samples of patients randomized to treatment B (n=123), in order to assess whether the treatment B was able to induce changes in gut microbiome diversity and composition. Kruskal-Wallis test showed significantly higher bacterial evenness at V4 versus V2 (Supplementary figure 7A, p=0.008). Richness analyses based on Faith PD diversity and observed OTUs index also showed no difference across the different time points (Supplementary figure 7, all p n.s. ) whilst diversity analyses using the Shannon index identified higher bacterial diversity in microbiome of patients at V4 versus V2 (Supplementary figure 7B, p=0.02).

**Alpha diversity between treatment arms.** To evaluate differences in gut microbiome between the two treatment arms at 21 days (Visit V4), we performed diversity microbiome analyses on samples (n=62) comparing the diversity results between the two treatments. Bacterial evenness and Shannon diversity resulted significant higher in treatment B versusA (Supplementary figure 8A-B, Kruskal-Wallis test p=0.027 and p=0.021, respectively). Bacterial richness calculated using faith PD and observed OTUs index showed no significant difference between treatments (Supplementary figure 8C-D, p=0.57 and p=0.4, respectively). Alpha diversity analysis between the two treatment arms at V5 showed no significant differences as well.

*Beta diversity analysis*

**Diversity analysis.** As first analysis we explored the composition of all sequenced samples to evaluate the quality of microbiome data. All sequenced samples ere made of fecal samples of the patients in the trial, positive controls (fecal samples from healthy individuals) and negative controls (samples generated during DNA extraction and library preparation). Diversity analysis can identify putative clusters and features inside the sample population. Principal coordinator analysis using different beta diversity metrics (unweighted unifrac, weighted unifrac, Bray Curtis) rarefying the samples at 14000 reads are reported in Supplemental Figure 9. Different bacterial metrics identified different sample distribution. Unweighted unifrac distance 9A revealed two separated clusters that are not visible using the other two metrics. We investigated the cause of this separation (Supplementary figure 9A). We nominated the upper cluster of the plot as "positive" and the lower as "negative". The positive cluster is made of 214 samples (24positive controls, 4 negative controls, 186 patients), whilst the negative cluster is made of 65 samples (12 positive controls, O negative controls, 53 patients). Since both clusters are made of samples from patients and positive controls , we investigated which technical or clinical' parameter could be involved in the generation of these two clusters. First, to assess if there was a batch effect among sequencing runs, we performed a Pairwise PERMANOVA test of unweighted unifrac distance matrix on all samples on the basis of sequencing runs (Supplementary figure 9) (279 samples made of 239 patients, 36 healthy control, 4 negative controls). The test highlighted significant differences among the sequencing runs (n=279, p<0.001).

However, the sequencing run did not seem to affect the distribution of the samples into the two clusters, and samples sequenced in the run)-2020 belonged to both clusters, and samples sequenced in different runs falled together inside the positive cluster. In addition, our analysis identified that the clinical parameters (concomitant treatment, antibiotics use 7 days before visit, number of evacuations, height and weight percentile of patient, etc.) did not affect the sample distribution in the PCoA. A sample contamination appeared to have happened in samples belonging to the negative cluster (Supplementary figure 10C), for this reason we decided to run alpha diversity analysis on the patient's samples belonging to the positive cluster.

*Alpha diversity and beta diversity of positive cluster*

**Alpha diversity comparison at baseline.** We performed also alpha diversity analysis on fecal samples belonging to the positive cluster. The analyses confirmed the alpha diversity results described above for all samples, showing no significant differences between the patients randomized tothe two treatment arms: treatment A n=44 and treatment B n=43 (Supplementary figure 11, n=87, p n.s.). In addition, we performed evenness analysis of patients at V2, grouped based on sequencing run and age. Sequencing run identified no significant differences among them (Kruskal-Wallis test, p n.s., Supplementary figure12A). Instead, significant differences were found grouping the samples on the basis of age (Supplementary figure 12B, Kruskal-Wallis test p<0.05). As expected, based on the literature, evenness increases with the increase of age. Similar results were observed performing diversity richness of bacterial species using Faith PD tree index (Supplementary figure 12C-D). Shannon and observed OTUs indexes reported similar results to Faith PD tree analysis (data not shown). The bacterial richness showed difference based on sequencing run (Supplementary figure12C) and increases according to age (Supplementary figure 12D), indeed patients within 6-12 months who were starting to introduce solid food showed the lowest index of bacterial diversity. These diversity results suggested that the age of patients and the sequencing run could affect the results during microbiome analysis.

**Alpha and beta diversity during treatment A.** We also performed diversity microbiome analysis exclusively on fecal samples of patients belonging to the cluster "positive", randomized to the treatment arm A (n=94). The analyses were performed comparing the gut microbiome of patients at baseline (V2) and after 21 days (V4) and after 56 days (V5). Principal coordinator analysis of unweighted unifrac distance revealed no significant difference of fecal samples collected at different visits (PERMANOVA test, n=94, p n.s., Supplementary figure 13A). Also, alpha diversity analysis performed on bacterial evenness (Kruskal-Wallis test) and on bacterial richness with Faith PD tree index (Kruskal-Wallis test) did not show any difference in the fecal samples before and after the treatment (Supplementary figure 13B-C, all p n.s.). Only alpha diversity calculated with Shannon index identified a significant increase of bacterial diversity at V5 (Kruskal-Wallis test, V2 vs V5, p=0.04). In addition, the age of the patients (Group 1, 6-12 months) appeared to influence both beta bacterial diversity (PERMANOVA test, n=94, p<0.01) and bacterial richness (Kruskal-Wallis test, n=94, p<0.01), as described in the population at baseline, while sequencing run influenced only bacterial diversity (PERMANOVA test, n=94, p<0.01) (Data not shown).

**Alpha and beta diversity during treatment B.** Principal coordinator analysis of unweighted unifrac distance of beta diversity revealed non significant differences of fecal samples collected during V2, V4 and V5. (PERMANOVA test, n=97, p n.s. , Supplementary figure 14A). Significant differences were observed , as expected, among sequencing runs (PERMANOVA test, n=97, p<0.05) and groups of age (p<0.01). Although the categories, sequencing run (Run1_2018 vs Run1_2020) and group of age (6-12months vs others), showed a significative effect on bacterial evenness (Kruskal-Wallis test p<0.01 and p<0.05, respectively), diversity analysis revealed that treatment B significantly increased bacterial evenness comparing gut microbiome samples collected at V2 versus V4 (Krustal-Wallis, n=97, test, p=0.004, Supplementary figure 14B). Bacterial richness analyses based on faith PD and observed OTUs indexes did not identify any effect of treatment B on microbiome (Supplementary figure 14C), while bacterial diversity analysis performed with Shannon index identified a significant increase of alpha diversity in gut microbiome of patients at V4 (Krustal-Wallis test, V2 vs V4, p<0.05, Supplementary figure 14D). The Shannon results were not surprising and were in agreement to evenness results, since Shannon index measures the number of species and scaled them on the evenness of the community.

**Alpha and beta diversity between treatment arms.** We evaluated the differences in gut microbiome between the two treatment arms on samples of patients belonging to the positive cluster collected at V4 (n=46).

Principal coordinator analysis of unweighted unifrac distance identified no difference between the two treatment arms at V4 (PERMANOVA test, n=46, p n.s. Supplementary figure 15A). Significant differences, as previously reported, were observed on the basis of sequencing run (Run1_2018 vs Run2_2018, PERMANOVA test p<0.05) and age group (Group 1, 6-12 months, vs others p<0.05). Instead, evenness and richness of samples at V4 analyzed per sequencing run and age groups did not show any significant difference (Kruskal-Wallis test, evenness, all p n.s. ; richness Faith PD all p n.s , data not shown).

Evenness diversity analysis highlighted a significant increase of bacterial evenness in the patients treated with treatment B as compared with patients treated with A (Kruskal-Wallis test, n=46, p=0.018, Supplementary figure 15B) confirming the results obtained analyzing all samples. No differences in bacterial richness (with Faith PD tree) were observed (Kruskal-Wallis test, n=46, p n.s. , Supplementary figure 15C) while, a significant increase in Shannon bacterial diversity was observed in patients under treatment B (Kruskal-Wallis test, n=46, p<0.05, Supplementary figure 15D).

These results highlighted a strong and significant difference in the effect of the two treatments. This different effect on gut microbiome diversity was able to overcome the strong contribution of patient's age and batch effect due to different runs. Diversity analyses of fecal samples both of all samples and of the positive cluster did not show any significant difference between the two treatments (data not shown). Only age groups (group 1, 6-12 months against other age) showed significant differences in richness and diversity (Kruskal-Wallis test, p<0.05). These results confirmed the alpha diversity analysis of all patient's samples.

*Gut microbiome taxonomy*

**Bacterial taxonomy at baseline.** We investigated the taxa composition of fecal samples belonging to the positive cluster collected at baseline grouped per age (Group1 6-12nnonths; Group2 12-24 months; Group3 24-36 months; Group4 more than 36 months). Taxa summary results revealed that the composition of gut microbiome during childhood depends on the group of age. These results are in accordance with literature and diversity analysis (described above). We plotted the relative abundance data of the top 15 dominant families (Supplementary figure 16). We observed correlation between group of age and some bacterial families. For example an inverse correlation is present between age group and Enterobacteriaceae family (at the increment of age we observed a decrement in the family), while the family of Lachnospiraceae, Ruminococcaceae increase with age group (Supplementary figure 16A).

Enterobacteriaceae are bacteria associated to infant age (unti) 12-14 months), while Lachnospiraceae and Ruminococcaceae, that belong to the phylum of Firmicutes, are associated to an adult-like microbiome, which presents bacteria able to breakdown a complex diet, rich in fiber and carbohydrates. Surprisingly, bacteria belonging to the phylum of Bacteroidetes, such as Bacteroidaceae and Prevotellaceae, which should appear during the weaning, were not detected. Furthermore, since the density of the microbiota, expressed as quantity of extracted DNA per mg of fecal samples, has been demonstrated to depend on physiologic features of the host and fitness of the gut microbiota, we normalized the relative abundance data for the quantity of extracted DNA per mg of fecal sample (Supplementary figure 16B). The absolute values highlighted a decrease of Enterobacteroidaceae, according to the age group, while Lachnospiraceae and Ruminococcaceae increased in group 2 (12-24 months) and then remained stable for the others group of age.

**Gut microbiome taxonomy during treatment A.** Taxa summary analysis of relative and absolute abundances on positive cluster were performed to evaluate the effect of treatment A on gut microbiome composition at different age groups (Supplementary figure 17A-B). Relative and absolute taxa results revealed that the treatment seemed to decrease Enterobacteriaceae family within the Group 1 and to increase the abundance of Bifidobacteriaceae and Ruminococcaceae families in all groups. Absolute abundance data (and not relative ones) showed an increment in Verrucomicrobiaceae family at V4 and a subsequent decrease at V5 for the groups 3 and 4.

**Gut microbiome during treatment B.** Taxa summary analyses on relative and absolute abundances were performed to evaluate the effect of treatment B on gut microbiome composition in different age groups (Supplementary figurel8A-B). Taxonomy results revealed that treatment B had a variable effect of Bifidobacteriaceae family depending on the age group. Bifidobacteriaceae decreased in Group 1 at V5, and increases in Group 2 and 3, while it seemed unaffected in Group 4.

**Comparison of microbiome composition.** In addition, to identify and compare changes in microbiome composition between the two treatment arms at genus level between V2 and V4 and between V4 and V5 we plotted the absolute abundance values in heatmaps. The results are plotted in the following supplementary figures based on age group.

**Group 1.** Changes in the abundance at genus leve! for age group 1 during the treatment A and B are showed in Supplementary figure 19. The heatmap showed interesting different features between the two treatments. Bifidobacterium spp, that is a fundamental genus at this age, increased during the different visits of treatment A, while in patients treated with treatment B they showed opposite trend by decreasing during the visits. Suttarella spp, Collinsella spp. and Dialister spp increased at V4 and then decreased at V5 during treatment A while were almost constant in treatment B. Also, Ruminococcus spp increased only in treatment A, while Akkermansía spp seemed to decrease only under treatment B between V2 and V4 and then to increase again during the follow up visit V5.

**Group 2.** Changes in the abundance at genus leve! for age group 2 during the treatment A and B are showed in Supplementary figure 20. The heatmap's results of group 2 identified features between the treatments that resulted different from group 1. Akkermansia spp presented a high abundance in group B and decreased during treatment B, between V4 and V5, in group 2 while in treatment A decreased between V2 and V4 and increased from V4 and V5. Two of the most abundant genera in age group 2 were Bifidobacterium spp and Escherichia spp. Interesting, in patients treated both with A and B Bifidobacterium spp decreased between V2 and V4, while Escherichia spp in group B increased between V2 and V4, but in both genera the abundance at V4 returned to the initial concentration at V2

**Group 3.** Changes in the abundance at genus level for age's group 3 during the treatment A and B are showed in Supplementary figure 21. Compared to the previous 2 groups, at this age children had a microbiota more similar to the adults, so some genera present in the previous groups decreased drastically to "give space" to new genera. Moreover, the heatmap appeared more confused as compared to the previous, and there were no evident changes. It was possible to observe an opposite effect in Akkermansia spp between A and B, but since the treatment B had a very higher starting abundance than treatment A the comparison was not possible.

**Group 4.** Changes in the abundance at genus leve! for age group 4 during the treatment A and B are showed in Supplementary figure22. The heatmap's results of group 4 identified features between the treatments. As for Group 11 Bifidobacterium spp. increased during treatment A. Akkermansia spp increased in treatment A at V4 and decreased during treatment B.
